# Studies of HVC Plasticity in Adult Canaries Reveal Social Effects and Sex Differences as Well as Limitations of Multiple Markers Available to Assess Adult Neurogenesis

**DOI:** 10.1371/journal.pone.0170938

**Published:** 2017-01-31

**Authors:** Olesya T. Shevchouk, Gregory F. Ball, Charlotte A. Cornil, Jacques Balthazart

**Affiliations:** 1 GIGA Neurosciences, University of Liege, Liège, Belgium; 2 Department of Psychology, University of Maryland, College Park, Maryland, United States of America; Pennsylvania State University, UNITED STATES

## Abstract

In songbirds, neurogenesis in the song control nucleus HVC is sensitive to the hormonal and social environment but the dynamics of this process is difficult to assess with a single exogenous marker of new neurons. We simultaneously used three independent markers to investigate HVC neurogenesis in male and female canaries. Males were castrated, implanted with testosterone and housed either alone (M), with a female (M-F) or with another male (M-M) while females were implanted with 17β-estradiol and housed with a male (F-M). All subjects received injections of the two thymidine analogues, BrdU and of EdU, respectively 21 and 10 days before brain collection. Cells containing BrdU or EdU or expressing doublecortin (DCX), which labels newborn neurons, were quantified. Social context and sex differentially affected total BrdU^+^, EdU^+^, BrdU^+^EdU^-^ and DCX^+^ populations. M-M males had a higher density of BrdU^+^ cells in the ventricular zone adjacent to HVC and of EdU^+^ in HVC than M-F males. M birds had a higher ratio of BrdU^+^EdU^-^ to EdU^+^ cells than M-F subjects suggesting higher survival of newer neurons in the former group. Total number of HVC DCX^+^ cells was lower in M-F than in M-M males. Sex differences were also dependent of the type of marker used. Several technical limitations associated with the use of these multiple markers were also identified. These results indicate that proliferation, recruitment and survival of new neurons can be independently affected by environmental conditions and effects can only be fully discerned through the use of multiple neurogenesis markers.

**Highlights**

- Endogenous and exogenous markers of new neurons differentially identify neurogenesis
- Thymidine analogues label neuronal populations born at specified moments
- Doublecortin gives an integrated view of neurogenesis changes over extended periods
- BrdU antibodies detect EdU-positive cells to a variable extent depending on their age
- Young and slightly older HVC neurons are differentially affected by social conditions

## Introduction

Adult neurogenesis was first discovered in the rat hippocampus [1], however, it was a series of experiments in songbirds that conclusively demonstrated the production, functional integration and electrophysiological activity of newborn neurons in the adult brain [2], triggering a new wave of interest in the phenomenon. Songbirds continue to be a useful model for the study of adult neurogenesis due to some unique features of the phenomenon in this taxon such as widespread migration of newborn neurons throughout the telencephalon, higher rates of proliferation than in mammals and the establishment of long-distance projections made by the newborn neurons in certain cases [3,4]. One specific neurogenic region, the song control nucleus HVC (used as a proper name), is of particular interest due to its important and specific role in the regulation of song behavior. By investigating the regulation of HVC neurogenesis, we can not only gain insight into the molecular and cellular aspects of this process, but also probe for the function of adult neurogenesis.

HVC is at the crossroad of three pathways involved in the learning, maintenance and production of song–the caudal motor pathway, the anterior forebrain ‘feedback’ pathway and the auditory pathway related to the perception of species-typical auditory signals. HVC is highly plastic and sensitive to a range of modulating factors including hormones and a variety of environmental stimuli. In seasonally breeding songbirds the volume of HVC during the breeding season is 1.3 to 3 times larger than during the non-breeding season (reviewed in [5]). Neurogenesis contributes importantly to this growth, although soma size also changes across season [6]. Neuronal proliferation takes place in the lateral ventricle [7]. The neuronal progeny then migrate along radial glia into the parenchyma [8] reaching HVC within 1–2 weeks. During this same period only about 50% of these newborn neurons will survive [9] and this survival rate is enhanced by testosterone [10] and estradiol [11]. Post-synaptic activity also enhances the new neurons survival during their first month of life once they have extended their axon to the nucleus robustus arcopallialis [12].

Traditionally neurogenesis is studied with the use of one of two thymidine analogues, [^3^H]-thymidine or 5-bromo-2’-deoxyuridine (BrdU) and there are a few cases when these two markers have been combined in the same study (see [13] for an example in birds). More recently endogenous markers that label specific phases in proliferation and neuroblast development have also been used. Both exogenous and endogenous markers of neurogenesis have advantages but also pitfalls (for a comprehensive review see [14]). For example, BrdU, considered the gold standard for measuring neurogenesis by many, only stays in the circulation for a limited period after injection (less than an hour for songbirds: [15]) and therefore labels only a limited population of newborn neurons that replicated their DNA during a short time window. Given that newborn neurons have critical periods to environmental influences [16], analyzing only one specific cohort of neuroblasts born at a specific time potentially leads to overlooking experimental effects on neuroblasts born at a different time. In addition, BrdU incorporates into all proliferating cell types and does not discriminate between newborn neurons, glia and endothelial cells unless an additional cell-type marker is used in combination, which is technically more challenging and can decrease the detection sensitivity. Increasing the dose or number of injections can also lead to DNA damage followed by DNA repair during which BrdU is incorporated [17], so that cells with newly repaired DNA can be falsely counted as newly born cells. This type of labeling is however rare with the doses and injection schedules that are generally used by most investigators and the vast majority of BrdU-labeled cells after a substantial survival time are in fact new neurons as attested by the fact that they co-express in high proportion markers of young neurons such as doublecortin [18,19].

There are equally important problems associated with the use of endogenous markers of neurogenesis such as calretinin, Ki67, PCNA, pHH3, PSA-NCAM and doublecortin. Although several of these endogenous markers have still not been used in birds due to the lack of antibodies that cross-react with the avian antigens, doublecortin (DCX) has been validated as a marker of neuroblasts in neurogenic regions of the songbird brain [20]. DCX labels newborn neurons that present two distinct types of morphology–fusiform DCX cells that are presumably young migratory neurons and round DCX cells that likely represent neurons in the early differentiation stage [20,21]. At the sub-cellular level, DCX plays a role in controlling the polymerization of microtubules and stabilization of the cytoskeleton [22–24] both of which are important for the migration of young neurons [21,25]. However these mechanisms are also involved in reorganization of the dendritic arbor, neurite outgrowth and synaptogenesis [26–28]. Thus neurons undergoing these processes could also express DCX, but it has been demonstrated that DCX-positive cells found in neurogenic regions are in the vast majority of cases newborn neurons. For example, in rats that have been injected with BrdU for 12 days, 90% of DCX-immunoreactive cells in the dentate gyrus are strongly positive for BrdU and therefore are very likely to be newly born neurons [29]. In canaries injected with BrdU twice a day for 5 days, over 70% of doublecortin-positive neurons in HVC co-label for BrdU 10 days after the first injection [18,19]. Between 10 and 30 days post-BrdU injection the proportion of BrdU-positive DCX-immunoreactive neurons displaying a fusiform morphology decreases while the proportion of round DCX neurons increases, suggesting that indeed the fusiform phenotype is the more immature form that later develops into round DCX neurons.

The social environment profoundly influences the behavior and physiology of songbirds [30–32]. For example, in several species it has been shown that the presence of a female greatly reduces a male’s song output [33–35], while removal of the female reinstates high levels of singing [36]. Although there is evidence that singing activity per se has a positive feedback effect on HVC neurogenesis [37,38], one study from our laboratory indicated that males housed with a female have a larger volume of HVC than their more actively singing counterparts housed with another male [39], suggesting either a stimulatory effect of the female or an inhibitory effect of the male presence. Additionally, males housed with a female sing less than males housed in isolation, yet their density of newborn neurons in HVC is higher [35]. In zebra finches a rich social environment increases newborn neuron survival in HVC, Area X and the caudal nidopallium [40]. In the latter region younger newborn neurons (40 days) survive more after a larger change in the social environment, while slightly older neurons (60 days) have a higher survival following a mild social change [41,42]. Together these data suggest that in songbirds, just as in mammals [16,43], newborn neurons have critical periods during which their survival is sensitive to different environmental stimuli.

On the other hand, in many temperate zone songbird species, including canaries, females rarely sing and have smaller song control nuclei, including HVC, than males [44] even though the presence of song in females seems to be an ancestral feature [45]. Although female canaries are frequently used as a model to study the activational effects of testosterone on song behavior and growth of song control nuclei in adults in response to testosterone [46] including HVC neurogenesis [10,47], few studies have directly compared the male and female HVC volumes and neurogenesis in the same study. A sex difference in HVC volume persists in canaries when both sexes are treated with the same amount of exogenous testosterone [48]. Similarly, male starlings in the same hormonal condition as females continue to have a larger HVC volume but a lower density of newborn neurons [49]. Other studies have compared the neurogenesis in males and females in different endocrine conditions. Male canaries had a higher density of fusiform DCX-positive (DCX^+^) neurons than females in all photoperiodic conditions that they experience during an annual cycle, i.e., irrespective of whether they are photosensitive, photostimulated or photorefractory [18]. Male brown-headed cowbirds and red-winged blackbirds in breeding condition had larger HVCs and a lower density of DCX^+^ neurons than the females of their species [50].

To investigate the mechanism mediating the sex differences and the social effects on HVC volume and neurogenesis, we compared here males housed in three conditions–alone, with a female or with a male. The females housed with a male were treated as experimental subjects as well as stimuli in order to simultaneously investigate sex differences in neurogenesis. Sex steroid concentrations were clamped at levels representative of breeding condition for each sex via subcutaneous Silastic™ implants filled with testosterone in males and with estradiol in females. This was done to distinguish between direct effects of the social conditions on HVC (female presence, male-related stress, …) and effects mediated by the possible activation by the female of the hypothalamo-pituitary-gonadal axis [51,52]. Given the limitations associated with the use of only one type of marker for labeling newborn neurons, we decided to use a combination of markers of neurogenesis to test whether a better understanding of the regulation of HVC neurogenesis by the social environment and sex of the bird could be gained through this approach. All birds were thus injected with two analogues of thymidine at different times points to label neuronal populations born at two different times and evaluate whether neurons of different ages are differentially sensitive to the social environment. We complemented this approach with a quantification of the endogenous marker of newborn neurons, DCX. Previous studies in mammals and birds have combined the use of two markers for labeling new neurons (e.g., [13,53,54], see [14] for discussion) but this is to our knowledge the first time that two exogenous and one endogenous marker are used simultaneously, in particular for analyzing neurogenesis in the songbird HVC, a model system characterized by a very intense neurogenesis (much more active than in the mammalian hippocampus) that provides more sensitive measures of changes in the neurogenesis process.

## Methods

### Animals

A total of 18 canaries of the Fife fancy breed were used in this study. All birds were in their second year; they were born and had gone through a full breeding cycle in the colony maintained at the University of Antwerp, Belgium during which they had been exposed to a minor immune challenge or its control manipulation and their body temperature had been recorded between 9 and 12 months of age (see [55] for details on the procedure and its effects). This manipulation was balanced across the groups formed for the current study so that it could not affect the group differences to be observed here. All experimental procedures complied with Belgian laws concerning the Protection and Welfare of Animals and the Protection of Experimental Animals, and this experimental protocol was approved by Institutional Animal Care and Use Committee (IACUC) called the Ethics Committee for the Use of Animals at the University of Liege (Protocol number 926). The canaries were exposed to a natural photoperiod for the months preceding their arrival in our lab at the University of Liege in September and were then housed in single sex groups of 9–10 subjects on short days (8L:16D) for 2 months before the start of the experiment to induce photosensitivity. One month after arrival (at the time of castration), it was confirmed that the males had regressed testes and were in a nonbreeding condition. Throughout their stay in the laboratory, all birds had *ad libitum* access to food (a mix of various seeds designed for canaries), grit, cuttlebone and water for drinking and bathing. A small amount of egg yolk food was additionally added approximately once a week. Their health status and food/water provision was checked daily including during weekends as required by the Belgian law on the use of experimental animals.

### Experimental procedures

Castration of all males was performed under general isoflurane anesthesia (3% for induction followed by 2–2.5% for maintenance) as described previously by Sartor and colleagues [56]. Each testis was removed through an ipsilateral incision during two surgeries separated by one week of recovery. Birds were then maintained under a warm lamp under visual inspection until they fully recovered from the anesthesia (a process that took only a few minutes) after which they were returned to their home cage. They were then checked several times during the next 24 hours to detect any possible problem. All subjects were observed to perch and feed within one hour. After both testes had been removed, males were allowed to recover for a minimum of two weeks. One day before being transferred to the experimental conditions, all subjects received a Silastic™ implant (Degania Silicone; internal diameter 0.76 mm, external diameter 1.65 mm, length 10 mm) which had been pre-incubated in 0.9% saline at 37°C overnight. For males, the Silastic™ implant was filled with crystalline testosterone, for females with 17β-estradiol (both Fluka Analytical, Sigma-Aldrich), in order to clamp the concentrations of these sex steroid hormones in both sexes to high values typical of the reproductive season. These experimental conditions were selected to allow us to separate direct effects of social conditions on neurogenesis from indirect effects that would result from a change in circulating concentrations of testosterone induced by the different social conditions. Females were treated with estradiol to ensure they would be receptive and thus provide optimal stimuli for the males. These implants have been shown to establish in the canary blood stable concentrations of testosterone or estradiol and activate morphological and behavioral responses that are typical of what is observed during reproduction for periods longer than 3 weeks (e.g., [46,48,57–60]).

One day after implantation of the Silastic™ capsules, subjects were moved from their group housing cages to their respective experimental social context and the photoperiod was changed from 8L:16D to 11L:13D. During that day subjects were also injected intraperitoneally with bromodeoxyuridine (BrdU, Fluka [Sigma Aldrich], ref no. 16880; 10mg/mL in 0.01M Phosphate Buffer Saline, PBS) 5 times with 2 hours between each injection, at a dose of 50mg/kg per injection. The first of these injections was given at the same time when birds were moved from their group housing to the experimental social conditions and other injections followed 2 hours apart. On day 12 of the experiment all subjects were injected 5 times with 5-Ethynyl-2´-deoxyuridine (EdU, Invitrogen, ref no. E10187) at a dose equimolar to the dose of BrdU, i.e. 41.07 mg/kg EdU in 0.01M PBS, following exactly the same injection schedule as for BrdU.

### Social context manipulations and behavioral observations

The social context manipulations consisted of housing birds in one of the three following conditions: testosterone-treated male housed alone (M; n = 3), testosterone-treated male housed with another male in the same endocrine condition, i.e. treated with testosterone (M-M; n = 6), testosterone-treated male housed with an estradiol treated female (M-F). In the M-F condition both birds served as experimental subjects; results of the male in the pair will be labeled M-F (n = 5) while results of female in the pair will be labeled F-M (n = 4 due to the loss on one brain). Care was taken to distribute birds from the same pre-experimental cage across different treatment groups and to ensure that birds housed together for the experiment were neither siblings nor members of a previously breeding couple. Due to time constraints, the experiment was run in 4 replicates that were started 2 days apart. Each replicate contained roughly equal numbers of birds in each of the 4 treatment groups. The subjects from one replicate were placed in adjacent cages to facilitate simultaneous behavioral observations. Subjects in one social condition were distributed randomly in the room. All subjects were in the same room in visual but not acoustic isolation from other cages. Final numbers of subjects in each group and for each dependent variable are indicated in all figures.

Every 2^nd^ day of the experiment starting from day 3, song rate of male subjects was quantified during a total of 10 min per day (total of 10 observation days). Each male subject was monitored for number of songs produced for 5 minutes in the morning and 5 minutes in the afternoon. During these 5 minutes, the observer sat quietly in front of the cages and noted the number of songs produced by each male. The different birds were observed each time in a different randomized order. We operationally defined song as a vocalization longer than approximately one second in duration after at least a 500 msec period of silence.

### EdU-BrdU cross-reactivity validation

The BrdU antibody we used (ABD Serotec, OBT0030, clone BU1/75) to quantify BrdU^+^ cells is known to cross-react with EdU [61] which could confound the detection of cells that incorporated only BrdU. To quantify the extent of cross-reactivity, we injected an additional four male canaries (obtained from a local breeder in Belgium) with EdU only at the same dose and following the same protocol as for other subjects in this experiment. Either 4 hours (n = 2) or 24 hours (n = 2) after the 5^th^ injection birds were killed by transcardial perfusion. The perfusion, brain collection, cryoprotection and processing followed the same protocol as for the other subjects in this study. One series of brain sections obtained from these subjects were labeled for BrdU and EdU.

### Blood collection and hormone measurements

Blood samples were taken from all subjects 4 to 7 days before the experiment (baseline), 4 days after the onset of social context manipulations, and during brain collection. 30–100 μL of blood was drawn from the brachial vein, in most cases within 3 minutes of catching. Samples collected between 3 and 4 minutes did not show increased corticosterone concentrations above baseline, therefore only a few samples collected more than 4 minutes after the initial bird capture were excluded from analyses of corticosterone. Blood samples were collected into heparinized micropipettes (Brand, Wertheim, Germany), transferred into Eppendorf™ microtubes and stored on crushed ice. Samples were centrifuged at 9000 g for 9 minutes, the supernatant plasma was collected and stored at -80°C until further use.

Before hormone enzyme immunoassays (EIA), steroids were extracted from the samples to remove potentially interfering compounds using liquid phase extraction. Spiked samples were always processed in parallel with the experimental samples to assess extraction recovery. Spiked samples consisted of the same type, amount and dilution of plasma as experimental samples but contained in addition 30,000 counts per minute (CPM) of the tritiated hormone of interest. Recovery rates (ratio of CPM recovered to CPM added) were used to correct all assay results.

10 μL of plasma was diluted in 150 μL of deionized water (MilliQ) in glass test tubes, samples were kept at +4°C for 30 minutes, and 2 mL of the non-polar organic solvent dichloromethane was added. Samples were vortexed and then left immobile for 1–2 hours to allow separation of the organic and aqueous phases. The organic phase was moved to a new test tube and dried under nitrogen gas at 40°C. The dichloromethane extraction was repeated a second time and pooled extracts were kept at -20°C until the EIA assay. Average recovery rates were 80% for testosterone and 84% for corticosterone. Testosterone and corticosterone concentrations were measured using EIA kits (Cayman Chemicals). On the day of the assay, samples were re-suspended in 400 μL EIA buffer from the kit (including 10% ethanol in the case of testosterone) and placed on a shaker set to 1350 rpm for 1 hour. The assay was performed immediately after following instructions provided with the kit. These assays have been previously validated for measuring these hormones in avian plasma [62–67].

### Brain collection and processing

After 21 days of exposure to the experimental conditions, all birds were deeply anaesthetized by an injection of 0.03–0.04 mL Nembutal™ (Sodium Pentobartbital 60mg/ml) and brains were collected from all subjects after transcardial perfusion. Blood was cleared from the brain by perfusion with 200 mL PBS 0.01M, followed by 200 mL 4% paraformaldehyde (PFA; 4.3 g/L NaOH, 40 g/L paraformaldehyde, 18.8 g/L NaH_2_PO_4_.H_2_0). The brain was extracted from the skull immediately after perfusion and post-fixed overnight in 15 mL PFA. The following day brains were cryoprotected in 30% sucrose (15.6 g/L Na_2_HPO_4_, 1.5 g/L KH_2_PO_4_, 300g/L sucrose) until they sunk to the bottom of their vial. Brains were then frozen on dry ice and stored at -80°C until further use. Brains were cut coronally into 4 series of 30 μm thick sections on a Leica CM 3050S cryostat and stored in anti-freeze (0.01M PBS with 10 g/L polyvinylpyrrolidone, 300 g/L sucrose, and 300 mL/L ethylene glycol) at -20°C.

### Nissl and immunohistochemical staining

#### Nissl staining and volume reconstruction

One series of sections was mounted on Superfrost slides, dried at least overnight, and Nissl-stained with toluidine blue. After differentiation in Walpole buffer and molybdate, they were dehydrated in a series of increasing isopropanol concentrations, in 99% ethanol and finally in xylene and coverslipped using Eukitt as a mounting medium. To reconstruct HVC volumes, photomicrographs were taken of each section in the series containing the nucleus, in both left and right hemispheres with a camera connected to an Olympus BH-2 light microscope at 4x magnification. An outline was drawn around the perimeter of each cross-section of the nuclei using ImageJ v1.47v (National Institutes of Health) and the delimited area was measured. When a section was missing, the area was estimated by taking the average of the two sections immediately rostral and caudal to it. The volumes of nuclei were calculated by summing the areas and multiplying by 120 μm, the distance between two successive sections in the series. The volume of the nuclei in each hemisphere was calculated separately and the average of the two measures was used for statistical analyses.

#### Doublecortin (DCX) and BrdU immunohistochemistry

**Fig 1:**
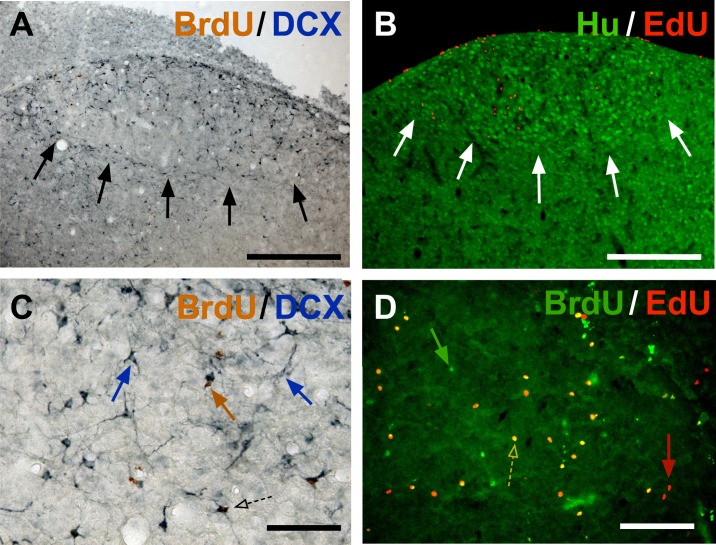
**Photomicrographs illustrating the different labels used to identify new neurons in HVC at low (A-B) and high (C-D) magnifications.** The first two panels show sections through the entire HVC that were stained for BrdU (brown) and DCX (blue; A) or with Hu (green) and EdU (red; B) illustrating the fact that these markers highlights the boundaries of HVC (arrows). Panel C shows at higher magnification a cell labeled for BrdU only (brown arrow), two DCX-positive neurons indicated by blue arrows, one round (left) and one fusiform (right) in shape) and one DCX-positive neuron also containing BrdU in its nucleus (dotted arrow). Panel D illustrates the double label for BrdU and EdU with arrows pointing to cells labeled for BrdU only (green), EdU only (red) and for both thymidine analogs (dotted arrow). Magnification bars are 200 μm in A-B and 50 μm in C-D.

A second series of sections was double-labeled by immunocytochemistry for BrdU and doublecortin (DCX). Sections were washed 3 times in Tris-Buffer Saline (TBS) 0.05M at the start and between each subsequent step except prior to primary antibody incubation. All sera and antibodies were diluted in TBST (TBS + 0.1% Triton-X100), while H_2_O_2_, avidin, biotin and diaminobenzidine (DAB) were diluted in TBS. Endogenous peroxidases were blocked using 0.6% H_2_O_2_ for 20 minutes, DNA was denatured to reveal the BrdU epitope in the chromatin by incubating the tissue in 2N HCl at 37°C for 20 minutes. The pH of the tissue was then neutralized during a 10-minute incubation in 0.1M sodium borate buffer. The non-specific binding of the secondary antibody was blocked by incubation in 10% donkey serum for 30 minutes and BrdU was labeled overnight with a primary rat anti-BrdU antibody (ABD Serotec, OBT0030) at a concentration of 1:2000. On the next day, sections were incubated for 2 hours with donkey anti-rat biotinylated antibody (Jackson, 1:2000), the signal was amplified by incubation for 90 minutes in ABC kit (Vectastain Elite PK-6100, Vector Laboratories), both components A and B being used at a concentration of 1:400. The BrdU antibody binding sites were revealed using 0.04% DAB in 0.012% H_2_O_2_ for 10 minutes, which produced a brown precipitate (Fig 1A and 1C).

For the second immunostaining sequence, sections were again incubated for 20 minutes in 0.6% H_2_O_2_, blocked for non-specific binding of the second biotinylated antibody by incubation for 15 minutes each, first in avidin then biotin solution, both at a concentration of 1:5. Sections were blocked in 10% rabbit serum and incubated for 2 hours at room temperature and then for two days at 4°C in goat anti-DCX (Santa Cruz, sc-8066) at a concentration of 1:200. Next, sections were incubated with 1:100 biotinylated rabbit anti-goat antibody for 2 hours, for 90 minutes in ABC kit as previously, and finally revealed for the DCX antibody binding sites with Vector SG Substrate kit (Vector laboratories, 15 μL chromogen and 24 μL H_2_O_2_ per mL of TBS) for 6 minutes, which produced a blue-gray precipitate (Fig 1A and 1C). Sections were mounted on glass slides and coverslipped using Eukitt (Sigma-Aldrich) as a mounting medium.

#### Hu immunohistochemistry combined with EdU Click-IT reaction

The Click-IT kit (Invitrogen, Catalog number C10338, using Alexa Fluor®555 azide, 555/565 nm excitation/emission, as fluorochrome) was used to label cells that incorporated EdU. In order to delineate the HVC area, this was combined with immunohistochemistry for Hu, a neuronal marker that has been shown in songbirds to be expressed specifically by neurons starting soon after their birth [68]. Many neurons in HVC are larger and denser than in the surrounding nidopallium and therefore the Hu-staining can be used to visualize the borders of HVC (Fig 1B). EdU-labeling was performed according to the instructions of the kit and Hu immunohistochemistry was started immediately after. PBS 0.01M and PBST 0.1% were used and 3 washes followed each step. The epitope was unmasked by incubating sections in citrate buffer (2.1g/L citric acid, adjusted to pH 6 using 1N NaOH, 0.5 mL/L Tween 20) for 2 hours at 37°C. The primary antibody incubation (1:100 mouse anti-Hu, Invitrogen, A-21271) was combined with 10% normal goat serum blocking and was performed for 2 hours at room temperature followed by overnight incubation at 4°C. Sections were then incubated with biotinylated goat anti-mouse secondary antibody at a concentration of 1:100 for 2 hours at room temperature. Finally, sections were incubated with streptavidin conjugated to Alexa Fluor 488 at a concentration of 1:100 for 90 min at room temperature. After the final wash, the sections were mounted on glass slides and coverslipped using Vectashield with DAPI as a mounting medium.

#### BrdU immunohistochemistry combined with EdU Click-IT reaction

EdU-labeling was performed according to the instructions provided by the manufacturer and BrdU immunohistochemistry was started immediately after. PBS 0.01M and PBST 0.1% were used for washes and antibody/serum dilutions. The sections were washed 3 times at the start to remove the antifreeze and following each incubation. DNA was denatured to reveal the BrdU epitope in the chromatin by incubating the tissue in 2N HCl at 37°C for 20 minutes. The pH of the tissue was then neutralized during a 10-minute incubation in 0.1M sodium borate buffer. The non-specific binding of the secondary antibody was blocked by incubation in 5% goat serum with 1% bovine serum albumin for 60 minutes and BrdU was labeled overnight at 4°C with a primary rat anti-BrdU antibody (ABD Serotec, OBT0030) at a concentration of 1:500. Sections were then incubated with biotinylated goat anti-rat secondary Alexa Fluor-488 antibody at a concentration of 1:500 for 2 hours at room temperature (Fig 1D). After the final wash, the sections were mounted on glass slides and coverslipped using Vectashield with DAPI as a mounting medium.

### Microscopy

#### BrdU and/or DCX-positive cells in HVC

A representation of the HVC borders was drawn on paper with the help of a camera lucida and a symbol was added on the drawing for each labeled cell, categorized as following: BrdU^+^DCX^-^, BrdU^-^Fusiform-DCX^+^, BrdU^-^Round-DCX^+^, BrdU^+^Fusiform-DCX^+^ and BrdU^+^Round-DCX^+^. The numbers of the different cell types were summed up for each HVC and the procedure was repeated on both sides of the brain and for 3–4 sections containing HVC per subject (except for 1 female where only 1 section with HVC could be counted). The total from both hemispheres was then averaged for all sections of a given subject and these mean values per section were used for analysis (see Statistical analysis). In addition, the BrdU^+^ cells in the ventricular zone (VZ) dorsal to each of these HVCs were quantified separately, as well as the length of this segment of VZ. For these counts, we only considered cells that were entirely included in the thickness of the VZ and ignored labeled cells as soon as their perikaryon had migrated out of this periventricular cellular layer.

#### EdU-Hu

EdU^+^ cells were quantified with a Leica fluorescence microscope (Leica DMRB FL.100; excitation filter BP545/30, dichromatic filter 565, suppression filter BP10/75) connected to a digital camera (Leica DFC 3000G). A photomicrograph was taken of each HVC, as detected by the dense group of Hu^+^ cells (observed with excitation filter BP480/40, dichromatic filter 505, suppression filter BP 527/30), in one series at 5x magnification and was used to quantify both the area of the nucleus and the number of EdU^+^ cells. The area was delineated based on the limit of the brighter, larger somas of neurons in HVC compared to surrounding nidopallium. EdU^+^ cells were counted manually in the entire cross section of HVC on these photographs in 3 to 6 sections per subject (except two females and one male with 1–2 sections of HVC counted). The EdU^+^ cells in the VZ dorsal to each of these HVCs were quantified separately, as well as the length of this segment of VZ, as described for BrdU and DCX cells.

#### EdU-BrdU

To quantify EdU^+^ and BrdU^+^ cells, the brain sections were visualized with a Leica fluorescence microscope (Leica DMRB FL.100; for EdU see filter specifications in 1.8.2; for BrdU: excitation filter BP480/40, dichromatic filter 505, suppression filter BP 527/30) and photomicrographs were taken with a digital camera (for cross-reactivity validation study—Leica DFC 480, for social context experiment—Leica DFC 3000G). In the cross-reactivity validation the EdU^+^ and BrdU^+^ cells in the VZ were counted on photomicrographs taken at 20x magnification on 4–6 sections from each brain. For the social context experiment brains, EdU^+^ and BrdU^+^ cells in one section of HVC were quantified on photomicrographs taken at 10x magnification.

### Statistical analyses

Densities of cells labeled by neurogenesis markers (DCX, BrdU, EdU) were obtained by dividing number of positive cells by the cross-sectional area of the HVC they had been counted in (number of cells/mm^2^). The density of cells/mm^2^ was corrected by the section thickness (30 μm) to obtain a density per mm^3^ (multiplied by 1000/30 = 30,3) and this density was then multiplied by the volume of the nucleus in mm^3^ to obtain an estimate of the total number in the entire nucleus. HVC volumes for this calculation were obtained from quantifications in Nissl-stained sections. These numbers that are extrapolated to the entire HVC provide a useful index to compare groups within the present experiment but should not be used for comparisons with independent studies since they are not absolute and depend on several parameters of the current study such as the section thickness and microscopic depth of field with the objective that was used, not to mention the staining efficiency.

The number of BrdU^+^ and EdU^+^ cells in the VZ was analyzed both as a number of cells in the entire VZ adjacent to HVC (per section, regardless of length) or as number of cells per mm of VZ adjacent to HVC. Although the VZ length per section was obviously different between males and females and possibly across male groups, since a large number of the new HVC neurons seem to originate in the adjacent VZ ([69] but see [4] for discussion), both analysis provide complementary information: data for the entire VZ relate more or less directly to the numbers of cells calculated for the entire HVC whereas data per mm of VZ relate to the densities within HVC.

A larger group of animals had initially been included in the experiment, however a subset of birds lost their Silastic™ implants during the experiment and thus had to be eliminated from the analysis. Additionally, in 3 males and 1 female the HVC was not complete in the sections that were collected because the caudal end of brain had been lost during processing. These subjects are excluded from the analyses that require the full extent of HVC, such as volume, but not other analyses, such as density of neurons where these markers were evaluated in only a subset of HVC sections. Extrapolation of these cells densities to the total numbers of cells in the entire HVC was obviously also impossible in these cases. The final numbers of data points available for each analysis are indicated in all graphs that also contain a representation of individual data points so that intragroup variability can be accurately appreciated. These small sample sizes obviously call for caution when interpreting the observed group or sex differences but they nevertheless allow us to establish the clear value of using multiple markers of neurogenesis in the same subjects. This approach should in the future be used more generally to dissect the time course of the production and incorporation of new neurons in HVC.

Analyses of the total numbers of different cell types in HVC were performed after either eliminating the subjects without a full HVC or after substituting the group means for these subjects. These two approaches yielded the same results, therefore only results of the former approach will be reported. In one female no BrdU^+^ cells were detected in the brain, thus this subject was excluded from all BrdU-analyses. For pre-experimental corticosterone measurements three male subjects were excluded because the volume of plasma collected was too small to be assayed reliably.

All behavioral and morphological measures including measures of neurogenesis were analyzed by non-parametric methods as the majority of data sets were not distributed normally, as determined by the Kolmogorov-Smirnov test. For each variable, the data from the three groups of males and one group of females is displayed side by side on a single graph for each measure, however the statistical analyses were performed separately. A Kruskal-Wallis ANOVA was used to compare the three groups of males and a Mann-Whitney U test to compare the males to the females in the M-F group. When the Kruskal-Wallis ANOVA indicated a significant difference between groups, a Dunn’s Multiple Comparisons post-hoc test was performed. All analyses of the three male groups were additionally performed taking the average data per male-male dyad rather than using individual data of the two males in the dyad, in order to test whether social interactions within a dyad had a major impact on the conclusion. The results obtained in these two approaches were very similar and therefore only the latter type of analysis is reported. Corticosterone plasma concentrations were analyzed by a repeated-measures ANOVA with social context/sex and time as factors and the Bonferroni procedure was used as a post-hoc test. Linear regression was performed to correlate the number of EdU^+^ and BrdU^+^ cells in the VZ in the cross-reactivity validation study. Statistical analyses were performed using GraphPad Prism (GraphPad Software Inc.) or STATISTICA (StatSoft). All data are presented by the mean ± standard error of the mean (SEM). Effects were considered statistically different if the p-value (two tailed for comparisons of two groups unless otherwise mentioned) for the analysis was equal to or lower than 0.05.

## Results

### Singing behavior

**Fig 2:**
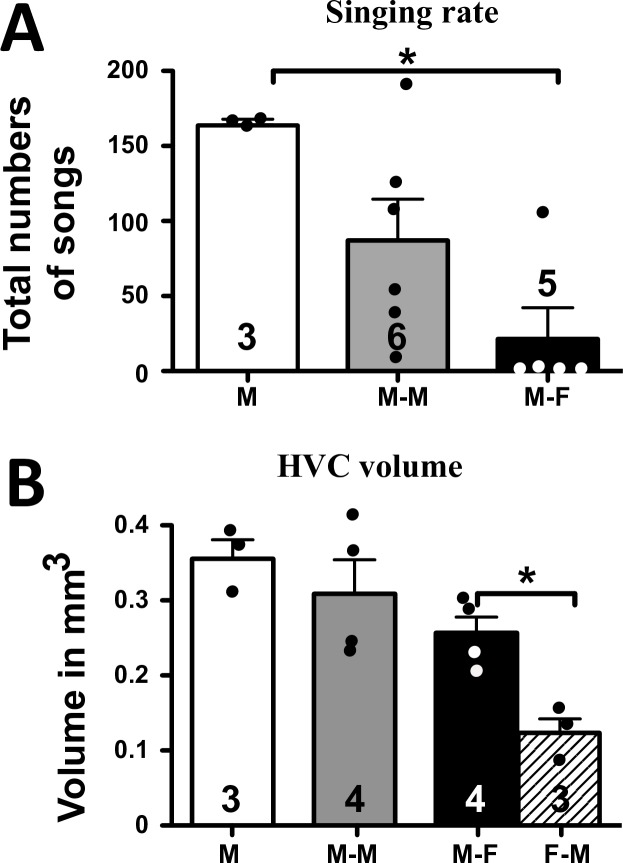
**Effect of social conditions on total number of songs measured during all observations (A) and on the volume of HVC measured in Nissl stained section (B). HVC volume in females is also shown.** M: male-alone, M-F: male housed with female, M-M: male housed with another male, F-M: female housed with a male. The figures on the bars indicate the numbers of available data. * = p<0.05.

Total numbers of songs recorded over the three weeks of observation were compared across the three male social conditions. The highest rate of singing was observed in male subjects housed alone (M) followed by males housed with another male (M-M) and then by males housed with a female, (M-F) (Fig 2A). Statistical analyses confirmed that the differences between groups were significant (H (df = 2, N = 14) = 8.251, p = 0.0162). Dunn’s Multiple Comparison post-hoc test showed that the male-alone group was significantly different from male with a female. Note however that a hypothesis-driven Mann Whitney test directly comparing the singing rates in the M-M and M-F groups suggested the existence of a significant difference also between these groups (U (N1 = 6, N2 = 5) = 3, one-tailed p = 0.015).

### HVC volume

As expected based on previous work [44,48] in the M-F dyads, HVC volume was bigger in males than in females (U (N1 = 4, N2 = 3) = 0, one-tailed p = 0.0286; Males: 0.257±0.021, Females: 0.122± 0.020, all means ± SEM; Fig 2B). HVC volume was not significantly different in males housed in different social conditions (H (df = 2, N = 11) = 4.303 p = 0.1161). The limited numerical differences between groups followed however the same general pattern as the differences in singing behavior (M > M-M > M-F).

### HVC neurogenesis

#### BrdU^+^ cells

**Fig 3:**
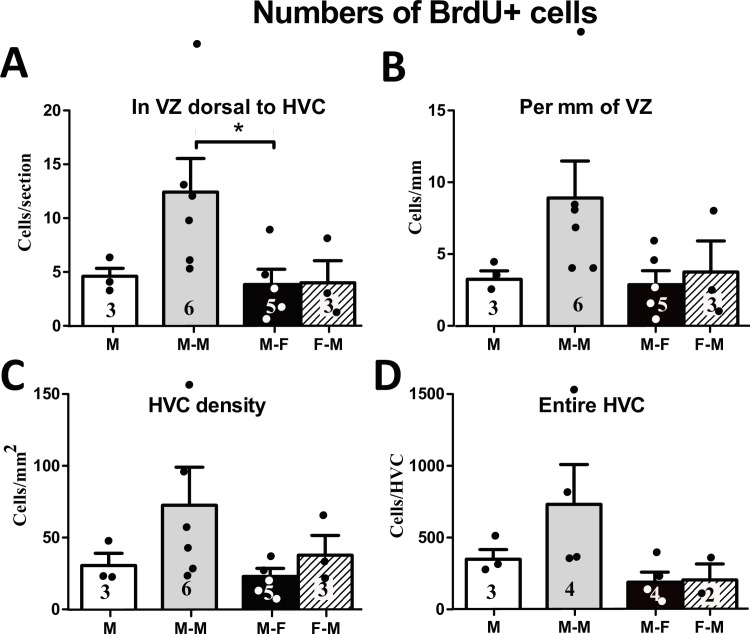
**Number of BrdU**^**+**^ **cells in the ventricular zone (VZ) dorsal to HVC as an absolute number per section (A) and normalized by the length of the VZ (B). Density (number per mm**^**2**^**) of BrdU**^**+**^ **cells in HVC (C) and number of BrdU**^**+**^ **cells estimated for the entire HVC (D).** M: male-alone, M-M: male housed with another male, M-F: male housed with female, F-M: female housed with a male. The figures on the bars indicate the numbers of available data points. * = p<0.05.

The overall number of BrdU^+^ cells in the VZ dorsal to HVC was significantly different between three groups of males subjected to different social conditions during 21 days (H (df = 2, N = 14) = 7.47, p = 0.024; Fig 3A). A post-hoc test showed that males housed with another male had more BrdU^+^ cells in the VZ than males housed with a female, the latter was not different from males housed alone. When the number of BrdU^+^ cells was normalized by the length of VZ, the difference in BrdU^+^ cells between the three groups of males was still significant (H (df = 2, N = 14) = 6.70, p = 0.035), however a post-hoc test did not reveal any pairs of groups that were significantly different from each other Fig 3B). Comparing the BrdU^+^ cells between males and females in the M-F group revealed no significant difference in terms of cells per section (U (N1 = 5, N2 = 3) = 7, p = 1.000), nor in terms of cells per mm of VZ (U (N1 = 5, N2 = 3) = 7, p = 1.00).

Inside HVC, the density of BrdU^+^ cells was also higher in male subjects housed with another male than the other two groups of males, but this difference did not reach significance (H (df = 2, N = 14) = 3.89, p = 0.143; Fig 3C). No sex difference in the BrdU^+^ density in HVC was found (U (N1 = 5, N2 = 3) = 5, p = 0.572). The estimated number of BrdU^+^ cells in the entire HVC was not significantly different across male subjects in different social conditions (H (df = 2, N = 11) = 4.55, p = 0.103; Fig 3D), neither was it different between sexes of equivalent social conditions (U (N1 = 4, N2 = 2) = 4.00, p = 1.000).

#### EdU^+^ cells

**Fig 4:**
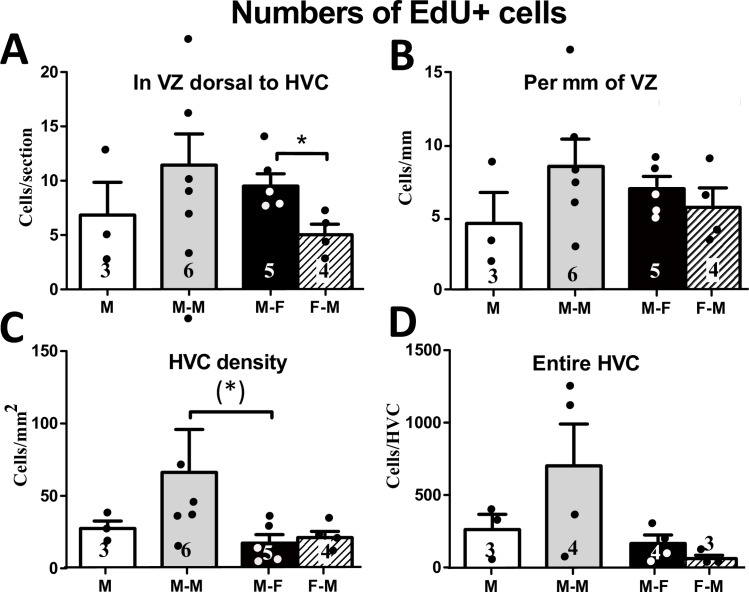
**Number of EdU**^**+**^ **cells in the VZ dorsal to HVC as an absolute number per section (A) and normalized by the length of the VZ (B). Density (number per mm**^**2**^**) of EdU**^**+**^ **cells in HVC (C) and numbers of EdU**^**+**^ **cells estimated for the entire HVC (D).** M: male-alone, M-M: male housed with another male, M-F: male housed with female, F-M: female housed with a male. The figures on the bars indicate the numbers of available data points. * = p<0.05; (*) = p<0.05 after an ANOVA showing only a statistical tendency (p = 0.053).

**Fig 5:**
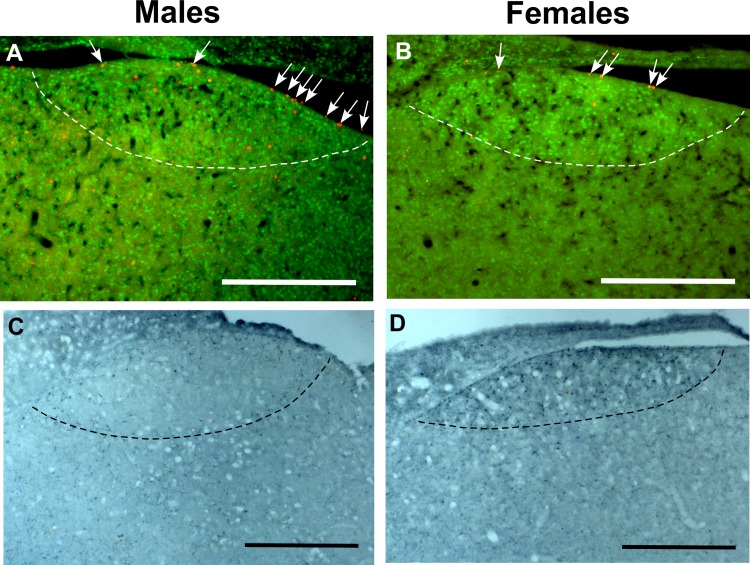
**Photomicrographs illustrating the sex difference in numbers of EdU+ cells in the VZ dorsal to HVC (A-B; Males >Females) and in the number of DCX+ neurons in HVC (C-D; Females>Males).** In panels A-B, EdU+ cells (white arrows) were labeled in red by the Click-IT reaction while the limits of HVC were identified by the higher density of Hu+ cells labeled in green. In all panels, the ventral edge of HVC is indicated by a dotted line. The magnification bars are set at 500 μM.

No difference between treatment groups was found in the number of EdU^+^ cells in the VZ dorsal to HVC in males (H (df = 2, N = 14) = 1.37, p = 0.504; Fig 4A). When the number of EdU^+^ cells in the VZ was normalized by the length of the VZ, no difference was still seen between different social conditions either (H (df = 2, N = 14) = 1.54, p = 0.463; Fig 4B). Comparison of sexes in equivalent social treatments (M-F vs. F-M) showed that females had fewer EdU^+^ cells per section (U (N1 = 5, N2 = 4) = 0, p = 0.016; Figs 4, 5A and 5B), but this difference disappeared when counts were normalized by the VZ length (U (N1 = 5, N2 = 4) = 0, p = 0.016; Fig 5B).

The density of EdU^+^ cells in HVC was marginally different between social condition groups in males (H (df = 2, N = 14) = 5.87, p = 0.053; Fig 4C) largely because males housed with another male tended to have a numerically higher density of EdU^+^ in HVC than males housed with a female (even if this difference is not significant). No significant difference was found in this measure between sexes in equivalent social conditions (U (N1 = 5, N2 = 4) = 8.00, p = 0.730). The estimated number of EdU^+^ cells in the entire HVC was not different between social conditions (H (df = 2, N = 11) = 2.91, p = 0.233: Fig 4D) neither between sexes in equivalent social conditions (U (N1 = 4, N2 = 3) = 2.00, p = 0.229).

#### Cross-reactivity validation

**Fig 6:**
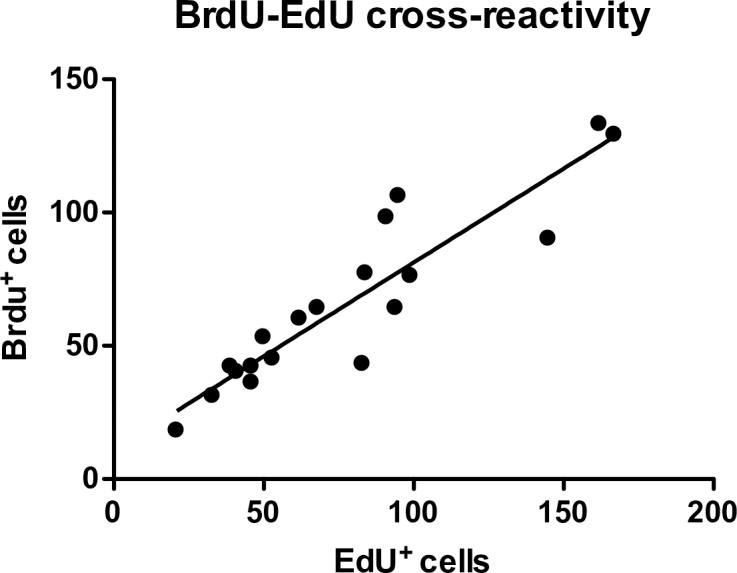
Linear regression of number of EdU-positive cells versus number of BrdU-positive cells in the VZ of the combined sections of birds injected with EdU only 4 and 24 hours before brain collection.

In male canaries injected with EdU only whose brains were collected 4 or 24 hours after the last injection there was a high degree of cross-reactivity of BrdU antibody (ABD Serotec, OBT0030) with EdU as shown by the correlation of the number of EdU^+^ and BrdU^+^ cells counted in segments of the VZ in the combined sections collected at both intervals after BrdU injections (r^2^ = 0.84, y = 0.70x + 10.79, Fig 6). The average percentage of EdU^+^ cells detected by the BrdU antibody was 87.6% (SEM = 3.7%, n = 19 sections). These data thus suggested that it should be possible to quantify both BrdU and EdU in sections from subjects injected with both thymidine analogs by subtracting from the BrdU-positive counts the numbers of EdU-positive cells.

#### BrdU-EdU co-labeling

Although the cross-reactivity validation showed that the BrdU antibody we used recognizes most EdU^+^ cells, the quantification of BrdU^+^ and EdU^+^ cells in the brains of subjects from the main experiments that had been injected with both markers identified some subjects with more EdU^+^ cells than BrdU^+^ cells. There were even social conditions where such a difference (more EdU^+^ than BrdU^+^ cells) was seen in the average counts (e.g. in the VZ of M-F males). This suggested that at least in some cases the cross-reactivity was not as complete as expected. To further investigate this question, we double-labeled for BrdU and EdU one section through HVC from each subject of the main experiment and quantified the single-labeled and double-labeled cells in the VZ dorsal to HVC and inside HVC.

Overall, the pattern of the single-labeled EdU^+^ and BrdU^+^ cells across groups was similar to the quantification of these labels performed on different sets of sections as summarized in Figs 3 and 4 (data not shown). Confirming our hypothesis regarding the lack of complete cross-reactivity in these brains, we observed that, on average, only 43.7 ± 0.06% (n = 18) of EdU^+^ cells in the VZ and 42.8% ± 0.07% (n = 18) of EdU^+^ cells in HVC were also labeled by BrdU. If we assume that some of these cells were BrdU^+^ because they had incorporated BrdU as well as EdU, the extent of cross-reactivity would be even lower. This is however unlikely since the BrdU and EdU injections were made 11 days apart.

**Fig 7:**
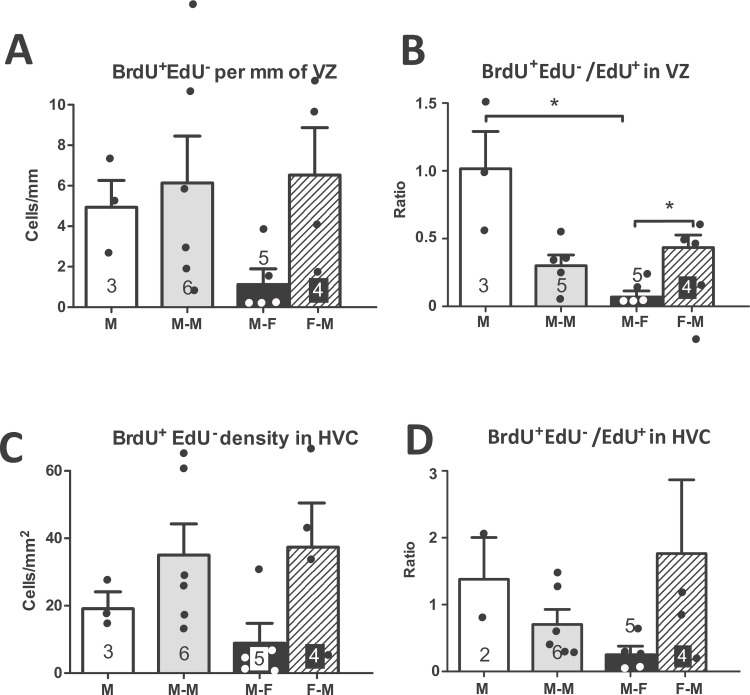
**Number of BrdU**^**+**^**EdU**^**-**^ **cells in the VZ dorsal to HVC per mm (A) and density (number per mm**^**2**^**) of BrdU**^**+**^**EdU**^**-**^ **cells in HVC (C). Ratio of BrdU**^**+**^**EdU**^**-**^ **over EdU**^**+**^ **cells in VZ (B) and HVC (D).** M: male-alone, M-M: male housed with another male, M-F: male housed with female, F-M: female housed with a male. The figures on the bars indicate the numbers of available data points. * = p<0.05.

Putting this methodological limitation aside, these counts of double-labeled cells allowed us to obtain an estimate of how many BrdU^+^ cells truly contained BrdU only (BrdU^+^EdU^-^) by subtracting from the total BrdU^+^ cells the number of BrdU^+^EdU^+^ cells. Although this number is possibly an underestimate because some of the EdU^+^BrdU^+^ cells could have incorporated both compounds (an unlikely event as discussed earlier in this section), this number still provides a useful estimate of how many of the cells born at the start of the social context manipulation and had incorporated BrdU at that time survived for 21 days. This analysis revealed that males housed with a female tended to have fewer BrdU^+^EdU^-^ cells than the other two groups of males both in the entire VZ (H = 5.32 (df = 2, N = 14), p = 0.070) and in HVC (H = 5.581 (df = 2, N = 14), p = 0.061; data not shown). This pattern was also present in the analysis of the density (numbers per mm or per mm^2^) of BrdU^+^EdU^-^ cells in the VZ (H = 5.51 (df = 2, N = 14), p = 0.064, Fig 7A) and in HVC (H = 6.43 (df = 2, N = 14), p = 0.093, Fig 7C). Comparing the males and females in the M-F group revealed a numerically larger number (VZ: U (N1 = 5, N2 = 4) = 3, p = 0.112; HVC: U (N1 = 5, N2 = 4) = 4, p = 0.190; not shown) and density (VZ: U (N1 = 5, N2 = 4) = 3, p = 0.112, Fig 7A; HVC: U (N1 = 5, N2 = 4) = 2, p = 0.064, Fig 7C) of BrdU^+^EdU^-^ cells in the brains of the females than in those of the males although these differences did not reach statistical significance.

We also compared across groups the ratio of BrdU^+^EdU^-^ cells over EdU^+^ cells as a measure of how many early born compared to late born cells survived. This comparison showed a significant difference between the three groups of males in the VZ (H = 9.44 (df = 2, N = 14), p = 0.009, Fig 7B) and a trend in HVC (H = 5.55 (df = 2, N = 13), p = 0.063, Fig 7D). Post-hoc tests indicated that males housed alone had a higher ratio of BrdU^+^EdU^-^ over EdU^+^ cells in the VZ than males housed with a female. Males housed with a female also had a lower ratio for the VZ than the females they were housed with (U (N1 = 5, N2 = 4) = 1, p = 0.032, Fig 7C) but this difference was not significant in HVC (U (N1 = 5, N2 = 4) = 3, p = 0.112, Fig 7D).

#### Doublecortin (DCX)

**Fig 8:**
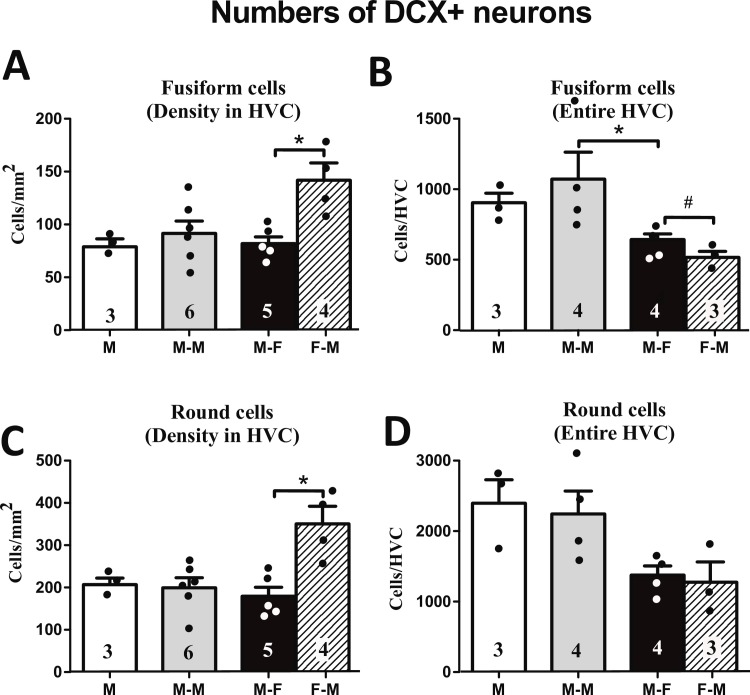
**Density of fusiform DCX**^**+**^ **(A) and round DCX**^**+**^ **neurons (C) in HVC. Numbers of fusiform DCX**^**+**^ **neurons (B) or round DCX**^**+**^ **neurons (D) estimated for the entire HVC.** The figures on the bars indicate the numbers of available data points. * = p<0.05, # = p<0.06.

The density of fusiform DCX^+^ neurons in HVC was not different between social groups (H (df = 2, N = 14) = 1.07, p = 0.586; Fig 8A). The comparison between sexes in the M-F group showed however a significantly higher density of fusiform DCX^+^ neurons in females compared to males (U (N1 = 5, N2 = 4) = 0, p = 0.016). A similar pattern was found for round DCX^+^ neurons, with no difference between social treatment groups (males: H (df = 2, 14) = 0.55, p = 0.759 and a significant sex difference with higher densities in females (U (N1 = 5, N2 = 4) = 0, p = 0.016; Fig 8C).

The estimated number of fusiform DCX^+^ neurons in the entire HVC was significantly different between the male social condition groups (H (df = 2, N = 11) = 7.05, p = 0.029; Fig 8C). A post-hoc analysis showed that males housed with a female had significantly fewer fusiform DCX^+^ neurons/HVC than males housed with another male. Males also tended to have a larger total number of fusiform DCX^+^ neurons in HVC than females (U (N1 = 4, N2 = 3) = 0, p = 0.056). There was a significant effect of social condition on the estimated total number of round DCX^+^ neurons for HVC in males (H (df = 2, N = 11) = 7.05, p = 0.029; Fig 8B), but the post-hoc analysis did not reveal any pairs of groups that were significantly different from each other. No sex difference was found in the estimated total number of round DCX^+^ neurons in HVC (U (N1 = 4, N2 = 3) = 5, p = 1.000).

### Hormone measurements

The blood samples collected at the end of the experiment from male subjects were assayed for testosterone concentrations, while samples from males and females collected at all three time points were assayed for corticosterone. Testosterone concentrations in the plasma of male subjects ranged from 1.24 to 6.84 ng/mL (mean = 3.56 ng/mL) and were not different between social conditions (H (df = 2, N = 14) = 1.07, p = 0.586).

**Fig 9:**
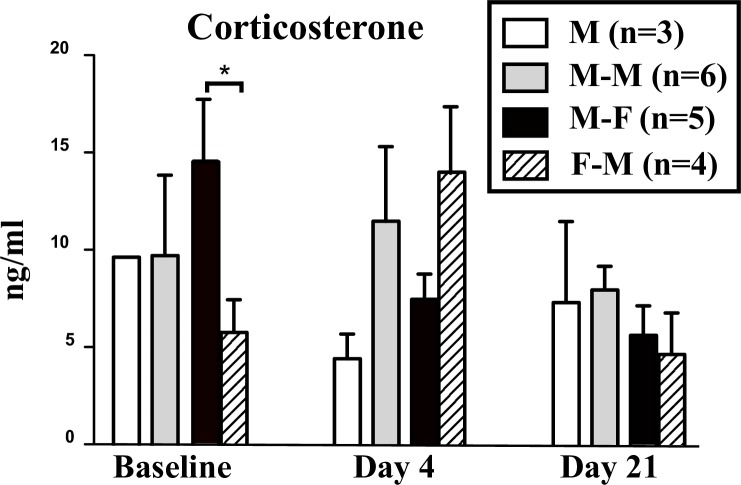
Plasma corticosterone concentrations measured in the 4 experimental groups before the experiment and after 4 and 21 days of exposure to the different social conditions. * = p<0.05.

A two-way repeated-measures ANOVA of corticosterone concentrations in the three male groups with time and social context as factors did not identify any main effects (time of sampling: F_2, 16_ = 1.90, p = 0.182, social context: F_2, 16_ = 0.36, p = 0.708) nor interaction between these factors (F_4, 16_ = 1.40, p = 0.278; Fig 9). The equivalent ANOVA comparing females and males in the M-F group identified no effect of time of sampling (F_2, 14_ = 2.75, p = 0.099), no main effect of sex (F_1, 14_ = 0.65, p = 0.448) but a significant interaction between the two factors (F_1, 14_ = 4.22, p = 0.037). Post-hoc analysis showed that before social context manipulations corticosterone concentrations were higher in males than in females. Interestingly, 4 days after onset of social conditions this pattern was reversed with females having a higher concentration of corticosterone than males, although this difference was not significant.

## Discussion

Most studies on adult songbird neurogenesis to have date employed a single proliferation marker. However due to the limitations of each marker, these investigations could be missing valuable information about the dynamics and regulation of neurogenesis. BrdU and other exogenous markers label small populations of cells born at specific times immediately after injections, doublecortin labels a broad population of neurons born over a large period before brain collection, although some disagreement exists concerning how long this labeling will last (see [14,69,70]). Exogenous markers are non-specific as regards the cell type that they label (in the brain they label both new neurons and glial/endothelial cells even if the former are more numerous, >70% of the total than the latter based on the co-localization with DCX; see [70] for discussion concerning the HVC of canaries) but largely label in a specific manner newly born cells. On the other hand doublecortin is neuron-specific but may also label neurons undergoing other types of plasticity. To exploit the advantages of both approaches, we combined here doublecortin and two exogenous markers, EdU and BrdU, to investigate effects of social context on HVC neurogenesis in male and female canaries. Each approach revealed substantially divergent patterns of neurogenesis as a function of the social condition or the sex of the birds.

### Methodological issues

EdU was introduced relatively recently and has proven to be very useful for studying cell proliferation (see for example [71–74]). Its detection, using a commercially available kit, is very specific, not labeling any other analogue of thymidine and compared to immunohistochemical staining of BrdU, the labeling is simpler, faster and the signal is stronger. Unfortunately, it is now becoming clear that this tool also has some drawbacks. Since our experiment was performed, a study comparing the survival time of cells labeled with EdU and CldU observed in mouse brain and in primary cultures exposed to EdU (but not to CldU) an increase in pyknotic cells and a decrease in EdU cells starting from 24 hours post-incorporation [53]. We noticed here that on the day after EdU injection, the injected birds were not looking healthy: their feathers were puffed up and some were sleeping with the head turned around in the night sleeping position. These organismal symptoms disappeared after 1–2 days but the cellular toxicity might have remained. Therefore, this marker should probably be used during *in vivo* studies only when the tissue of interest will be collected within 24 hours after injection.

An additional problem of EdU relates to its dose-dependent cross-reactivity with most anti-BrdU antibodies. Before initiating the main experiment presented here, a cross-reactivity test was performed with canaries that had been injected with EdU only; their brain had been collected 4 or 24 hours later and labeled for both EdU and BrdU. In this test, the cross-reactivity of our BrdU antibody (ABD Serotec OBT0030 rat monoclonal antibody) with EdU was 87.6%. Since this number was close to 100%, we assumed that we would be able to calculate the number of cells containing BrdU in sections of brains injected with both EdU and BrdU by subtracting the number of EdU^+^ cells detected from the number of BrdU^+^ cells detected. However, when the brains from the main study, which had been injected with both thymidine analogs 10 or 21 days before brain collection respectively were labeled for EdU and BrdU, the apparent cross-reactivity of the BrdU antibody with EdU was much lower: on average only 43.7 ± 0.06% (n = 18) of EdU^+^ cells in the VZ and 42.8% ± 0.07% (n = 18) of EdU^+^ cells in HVC were also labeled by BrdU.

This apparent decrease in cross-reactivity is possibly due to the fact that while at 24 hours most cells that had incorporated EdU had survived, 10 days later the cells that had incorporated a large amount of EdU had died and only those that had incorporated a smaller amount of EdU were still surviving. While the detection of EdU with the EdU kit is very sensitive and powerful, the detection of the low concentration of EdU in these cells by the BrdU antibody might give a weaker signal that was not detectable during quantification. An additional factor potentially decreasing the amount of EdU in cells at 10 days compared to at 24 hours post injection is that some cells had undergone their final division after multiple divisions of a progenitor that had incorporated EdU, each division further diluting the label.

The simultaneous use of these two thymidine analogs is thus bound to result in significant problems that relate to the toxicity of EdU, limiting its use to label cell proliferation just before tissue collection, and to its cross reactivity with most anti-BrdU antibodies [61]. We had also tried in preliminary experiments an anti BrdU antibody that has been claimed not to cross-react with EdU (antibody Exbio mouse monoclonal BrdU antibody, MoBu-1 clone, 11-286-C025: [61]) but this antibody was also very poor at detecting BrdU itself and had to be discarded. Until more specific BrdU antibodies have been developed or identified, we would thus suggest avoiding the simultaneous use of these two markers.

We assessed here for each marker both the density of positive cells as well as their estimated number in the entire HVC calculated based on the volume of this nucleus. In several cases a different pattern of group or sex differences was detected by these two approaches due to the contribution of the differences in HVC volume to the total count. Similarly, Yamamura and colleagues [75] found diverging effects of testosterone and its metabolites, 5α-dihydrotestosterone (DHT) and E2 alone or in combination, on total numbers of DCX neurons in HVC and on DCX^+^ neurons density in female canaries. These diverging effects on density and total number of newborn neurons in HVC probably relates to the fact that the growth of HVC is due not only to an increase in neuron number but also to changes in soma size and spacing of neurons. The two types of measures of neurogenesis are thus not necessarily correlated and this raises the question of which measure better relates to functional outcomes such as memory formation or production of specific song features. Although many interesting hypotheses have been proposed about the function of adult neurogenesis such as its role in song learning or song perception, a recent critical review (see [3]) has pointed out that the lack of clear causal links between the occurrence of adult neurogenesis and any behavioral or cognitive outcome suggests that the function of neurogenesis itself largely remains to some extent an open question (see [3]).

### Effects of social conditions

Compared to isolated males, the males housed with a female had a reduced rate of singing, a phenomenon that has been described in other songbird species including canaries [35]. In the current study males housed in a same-sex dyad also sang at rates that were intermediate between the M and M-F males. Although the difference of singing rates between M-M and M-F males was not significant in the general non parametric analysis, it became significant in a hypothesis-driven Mann Whitney tests focused on these two groups only, which thus replicates the result of a previously published study [39].

The number of subjects per group was reduced in the present experiment due to technical problems such as the loss of Silastic™ implants and this limited the statistical power of the study. However, the patterns of differences between groups clearly indicated that the way the three populations of newborn neurons labeled by BrdU, EdU and DCX were affected was not always equivalent. In a first step we quantified the BrdU and EdU labels separately on different sets of brain sections. Inside HVC both the EdU and BrdU density/total numbers per HVC tended to be higher in the M-M condition than in the other two groups even if group differences did not reach statistical significance in many cases (see Figs 3 and 4). This similarity probably reflects the fact that many of the BrdU-labeled cells are actually EdU cells detected by the BrdU antibody. Indeed, in the sections double-labeled for BrdU and EdU, almost one half of BrdU cells in HVC were also positive for EdU. This pattern seen in the total BrdU and EdU cells in the separate sets of sections was also observed when BrdU and EdU were co-labeled in the same set of sections (data not shown).

We additionally quantified BrdU and EdU cells in the putative site where many (most?) HVC new neurons are born: the VZ dorsal of HVC [76–78]. Although this measure does not represent the real rate of proliferation since large numbers of cells labeled at this location had plenty of time to migrate away and progenitors remaining in place could have lost a detectable signal over time after multiple divisions, it can serve as an indirect indicator of the proliferation rate at the time of injection or conversely as a negative measure of progenitor migration. Both the cells per section and cells per mm again showed a similar pattern for BrdU and EdU with the M-M group having more labeled cells than the other two groups, although in both measurements the effect was much less pronounced for EdU than for BrdU, thus providing a first suggestion that the cross-reactivity of BrdU with EdU cells does not completely explain the effect seen in BrdU. In the VZ of double-labeled sections approximately half of BrdU cells were also positive for EdU. These neurogenesis measures thus suggest a higher rate of proliferation and survival in males housed with another male, although this group also often displayed high inter-variability that prevented difference with other groups from being significant. This variability could be due to the dominance-hierarchy that was established in some pairs or alternatively reflect simply preexisting differences in birds assigned to the M-M group that potentially had a constitutively more active neurogenesis.

The double-labeled sections (BrdU and EdU) provided a more precise measure of cells that had incorporated BrdU by excluding from the total the BrdU cells that were also positive for EdU. The pattern for these BrdU^+^EdU^-^ cells (Fig 7) was somewhat different from the total BrdU^+^ or EdU^+^ cell counts (Figs 3 and 4). Males housed with a female had here a much lower number and density of these cells both in VZ and HVC than the other groups of males. Furthermore, when we assessed the number of BrdU^+^EdU^-^ cells relative to the number of EdU^+^ cells as a measure of relative survival of the older cells, the males housed with a female also had a lower ratio than the other groups, with males housed with another male falling in between the other two groups. These data thus suggest that in presence of a female, newborn cells that had been labeled by BrdU survived less than in other conditions. This effect can relate to migration, recruitment and/or survival but presumably not to a difference in proliferation since the BrdU labeling occurred when different social conditions had just been established for a few hours. The fact that this difference was not found in the count of EdU^+^ cells suggests that either social condition did not have enough time to affect these cells labeled only 10 days before brain collection or that an increase in proliferation compensated the decrease in recruitment/survival.

The density of fusiform or round DCX^+^ neurons was not different across social conditions in males, however the estimated number of fusiform and round DCX neurons for the entire HVC was numerically lower in males housed with a female than the other two groups of males, although this difference was significant only for the comparison of fusiform cells between the M-M and M-F groups (Fig 8B). In contrast, previous studies reported that males housed with a female have a higher density of DCX neurons than males housed alone [35] or males housed with another male [18]. Birds in both these studies were however maintained under a long-day photoperiod (16L:8D) while they were here on 11L:13D and in the former study birds were in acoustic isolation and exclusively affected by the social environment inside their cage, whereas the birds in the current and previous [18] study were visually isolated but could hear all other birds present in the same room. They could thus integrate acoustic cues from a large number of other birds and this complex acoustic environment could have partly masked some effects of the partner present in the same cage. These differences in design possibly explain why we saw a different pattern from previously published studies. Photoperiodic gating of the effect of social environment on HVC neurogenesis will be further discussed below.

Singing activity in M-M males was intermediate between activity in the M and M-F groups. Overall the ratio of BrdU^+^EdU^-^ over EdU^+^ in HVC and at the VZ level, and HVC volumes followed a similar pattern. The differential levels of singing between the groups could explain differences in some measures of neurogenesis. Singing has been shown to have a positive feedback effect on BDNF expression and neurogenesis in HVC [37]. It is interesting that this pattern is seen in measures of BrdU^+^EdU^-^ cells (presumably older neurons), of HVC volumes and to some extent in the numbers of DCX^+^ neurons in the entire HVC but not in the densities or total numbers of EdU^+^ cells (presumably younger neurons) nor in measures of total BrdU^+^ cells that are contaminated by EdU cross-reactivity. This would suggest that singing activity affects the survival of new neurons but only does so after a minimal amount of time that would, based on the current data, definitely need to be longer than 10 days. It has been suggested in mammals [16,43] and in zebra finches [41,42] that newborn neurons have a critical sensitive period when they are responsive to certain environmental cues. For example, work on the social context effect on neurogenesis in the caudal nidopallium of zebra finches indicates that changes in social conditions (transfer from a small group to isolation or to a large group) differentially affects cells labeled by BrdU if brain collection is performed 40, 60 or 150 days after labeling. It is thus possible that the EdU^+^ cells that were about 10 days of age at brain collection had not yet reached the sensitive period when the social context can affect their survival. Alternatively, the toxicity of EdU means that a large number of labeled cells must have died before brain collection and it is conceivable that those cells that survived constitute a sub-population that is less or not sensitive to the social environment.

Males housed with a female also had somewhat smaller HVC volumes and reduced levels of singing compared to other males, although this difference was only significant for the songs produced by males alone versus males housed with a female. There was also a lower rate of putative neuronal proliferation and survival in males housed with a female as detected by several measures which contrasts with previous studies that had identified effects in the opposite direction based on measures of HVC volumes or counts of DCX^+^ neurons [18,35,39]. Multiple factors could explain this discrepancy.

The effects of the social environment can indeed be complex. Our previous work showed that males housed with a female had a larger HVC than males housed with another male [18,39] but this experiment did not allow us to discriminate between an increase due to the female presence from a decrease due the presence of another male. Other studies have also found that males in acoustic isolation have a smaller HVC than males housed in group although the specific aspect of the social condition playing a key role have not been identified in a definitive manner [79]. The behavior of congeners including their vocalizations could for example be the determining variable here and more work should be done concerning the question of social effects on brain plasticity.

We also wondered whether the multiple manipulations performed in this but not in previous studies (blood sampling on day 4, multiple injections of BrdU on day 0 and of EdU injections on day 12, …) had potentially induced a stress preventing the positive effect of female presence. Assays of plasma corticosterone provided however no evidence for this interpretation: concentrations of this steroid were similar in the three male groups and, if anything, decreased in M-F males in the course of the experiment.

Another difference between this study and previous studies concerns the photoperiod the birds were exposed to. We kept birds under an 11L:13D photoperiod that insures they do not become photorefractory during the experiment, while previous studies identifying a positive effects of females on HVC were performed under an 16L:8D photoperiod [35,39,80]. It is possible that males become more sensitive to social cues related to reproduction and/or alternatively that females only start emitting positive signals conducive to enhanced neurogenesis under photoperiods mimicking spring and summer conditions. Moore showed [52] that male sparrows housed with females implanted with estradiol displayed an increased rate of mount attempts and higher testosterone and LH levels than males housed with non-sexually receptive females with empty implants when held on long-days but that under short-days males still mounted more the E2-treated females but no longer showed the increased testosterone and LH concentrations compared to the control males housed with sexually non-receptive females. In addition, the stimulatory effects of female presence on the development of the hypothalamus-pituitary-gonadal axis has been shown in quail to be effective only or more effective in long days [81]. On the female side, effects of estrogens on receptivity and on the response to stimulatory male songs are also influenced by the photoperiod. In ovariectomized canaries for example, exposure to male songs increases the estrogen-induced nest building only in marginally stimulating photoperiods (12L:12D). In short days nest building is not observed at all but in long days, activity is so intense that no further increase can be observed after exposure to male songs [32]. Similarly, in white-crowned sparrows, male song playback increases ovarian growth if females are in 12.5L:11.5D or in 14L:10D but not under 6L:18D or 11L:13D [82,83]. These studies provide evidence that neuroendocrine responses to behavioral cues can be dependent on the photoperiodic condition the bird experiences [83]. It is thus conceivable that under the photoperiodic conditions used here either females did not produce and send the adequate stimulatory stimuli to the males or males were not sensitive to these stimuli.

### Sex differences

The comparison of males and females in the M-F group also identified divergent patterns of sex differences as a function of the marker of neurogenesis employed. In HVC, the total numbers and the density of BrdU^+^ and EdU^+^ cells were both similar in the two sexes (Figs 3 and 4) and again, the similarity of these two sets of results probably reflects the cross-reactivity of the BrdU antibody with EdU. In contrast, however, the density of BrdU^+^EdU^-^ cells (Fig 7C), the ratio of BrdU^+^EdU^-^ to EdU^+^ cells (Fig 7D) and the density of both fusiform and round DCX^+^ neurons (Fig 7A and 7C) were higher in the female than in the male HVC.

However, when cell numbers were estimated for the entire HVC, the sex difference in round DCX^+^ neurons disappeared (Fig 7D) and the difference in fusiform DCX^+^ neurons was even reversed (males>females, Fig 7C). Together the data are thus supporting the fact that progenitor proliferation at the VZ and young neuron recruitment by HVC are overall very similar in males and females [84,85] even if small localized differences favoring females do exist in the rostral telencephalon [86] but since HVC volume is smaller in females, the density of these new neurons is larger in females than in males. Why this sex difference in density in favor of females was not detected in the analysis of EdU^+^ cells remains unexplained. It could be hypothesized that the cytotoxicity of EdU discussed in the previous sections could block migration of young neuroblasts away from the VZ thus causing a local accumulation and in parallel decreasing the density of EdU^+^ cells in HVC so that F-M values are equivalent to values in the M-F group (Fig 4C). Why this would affect females proportionally more than males is however unclear.

It is interesting to note that sex differences in HVC neurogenesis favoring females were detected with markers related to relatively older neurons (BrdU^+^EdU^-^ cells, DCX^+^ neurons) but not with EdU that was injected only 10 days before tissue collection and thus labeled comparatively younger cells. This sex difference concerning BrdU^+^EdU^-^ cells and DCX^+^ neurons but not EdU^+^ cells could be taken as evidence suggesting that female neurons are recruited and/or survive in HVC comparatively longer than male neurons. Unpublished data from our lab indeed suggested that newborn neurons in the HVC of females mature more slowly than in the male HVC, taking longer to down-regulate DCX [87]. This specialized difference related to older neurons could also potentially relate to the lower baseline corticosterone concentration in the females compared to males of the same M-F group. Since this difference was seen in baseline samples, before the establishment of differential social contexts, it has to reflect a pre-existing sex difference that later vanished in samples collected on days 4 and 21. Corticosterone decreases neural proliferation in mammals [88] and in male songbirds [89,90]. Exposure to a higher corticosterone concentration before but not during the experiment might thus explain the lower rate of neurogenesis in males compared to females observed in measures of older but not comparatively younger neurons.

The more active neurogenesis in females compared to males could seem at first sight counter-intuitive given that testosterone is known in males to increase new neurons survival [18,75,84,91–95] It must be recalled however that females in the present experiment were treated with estradiol and estrogens also increase neurogenesis in the canary HVC [11] while in female starlings, blocking estrogenic action decreases neurogenesis to the same low level as in males [49]. In agreement with the present study, female starlings, red-winged blackbirds and brown-headed cowbirds also had a higher density of DCX neurons in HVC than males [49,50]. This higher density of DCX neurons in females than males contrasts with results of a previous study where males had more fusiform DCX neurons and tended to have more round DCX neurons than females [18]. However, all the canaries in the present study were treated with exogenous hormones—testosterone for the males and 17β-estradiol for females—whereas birds in [80] were only exposed to their endogenous steroids. Although the photostimulated females in this previous study likely had relatively high levels of circulating estrogens, concentrations were not measured and they were possibly not as high as in estradiol-treated females of the present study. The discrepancy of results could also be due to the presence of gonads in males of the study of Balthazart et al [80] but not here, or to the different quantification approaches (counts in the entire cross-section of HVC here vs. counts in a 200 μm x 200 μm square in the center of HVC in [18].

Note also that the pattern of sex difference density per mm of BrdU^+^EdU^-^ cells in the VZ was similar to the pattern observed for the density of these cells in HVC (Fig 7A vs. 7C) suggesting that even 21 days after injections of the exogenous marker, the density of labeled cells in the VZ still reflects the initial proliferation rate despite the migration away of many labeled progenitors. This was however not true for the density of EdU^+^ cells per mm of VZ possibly due to the toxicity of this compound discussed before (Fig 4A vs. 4C). More work should clearly be devoted to understanding how EdU incorporated into cells replicating their DNA affects the subsequent survival of these cells.

## Conclusion

These results, despite their limited power related to small final sample sizes, demonstrate that the use of multiple markers is a very useful tool to understand the complexities of environmental influences on HVC neurogenesis. A limited number of endogenous markers have been validated for use in songbirds, including doublecortin that is particularly useful because it is neuron-specific and discriminates two different stages of neuroblast development, especially when combined with different analogues of thymidine which enable us to follow the trajectories of newborn neuron populations born at a specific time relative to the treatments administrated. Technical problems are however associated with the simultaneous use of multiple thymidine analogs including cross-reactivity in their detection and potential toxicity of EdU that should only be used as a marker of proliferation and injected less than 24 hours before brain collection. Even with these limitations, the present data suggest that proliferation, recruitment and survival of new neurons can be independently affected by environmental conditions with DCX providing cumulative information not necessarily reflected in measures of single new populations (BrdU^+^ or EdU^+^).

## Supporting Information

S1 Table Final full data set. (XLSX)

## References

[1] Altman J, Das GD. Post-natal origin of microneurones in the rat brain. Nature. 1965;207: 953–956. 5886931 10.1038/207953a0

[2] Paton JA, Nottebohm FN. Neurons generated in the adult brain are recruited into functional circuits. Science. 1984;225: 1046–1048. 6474166 10.1126/science.6474166

[3] Brenowitz EA, Larson TA. Neurogenesis in the Adult Avian. Cold Spring Harb Perspect Biol. 2015;7: 1–23. 10.1101/cshperspect.a019000 PMC4448602 26032719

[4] Balthazart J, Ball GF. Endocrine and social regulation of adult neurogenesis in songbirds. Front Neuroendocrinol. Elsevier Inc.; 2016;41:3–22. 10.1016/j.yfrne.2016.03.003 26996818

[5] Tramontin AD, Brenowitz EA. Seasonal plasticity in the adult brain. Trends Neurosci. 2000;23: 251–258. 10838594 10.1016/s0166-2236(00)01558-7

[6] Tramontin AD, Brenowitz EA. A field study of seasonal neuronal incorporation into the song control system of a songbird that lacks adult song learning. JNeurobiol. 1999;40: 316–326. 10440732 10.1002/(sici)1097-4695(19990905)40:3<316::aid-neu4>3.0.co;2-s

[7] Alvarez-Buylla A, Theelen M, Nottebohm F. Proliferation “hot spots” in adult avian ventricular zone reveal radial cell division. Neuron. 1990;5: 101–109. 2369518 10.1016/0896-6273(90)90038-h

[8] Alvarez-Buylla A, Nottebohm F. Migration of young neurons in adult avian brain. Nature. 1988;335: 353–354. 10.1038/335353a0 3419503

[9] Kirn JR, Fishman Y, Sasportas K, Alvarez-Buylla A, Nottebohm F. Fate of new neurons in adult canary high vocal center during the first 30 days after their formation. J Comp Neurol. 1999;411: 487–494. 10413781

[10] Goldman SA, Nottebohm F. Neuronal production, migration, and differentiation in a vocal control nucleus of the adult female canary brain. Proc Natl Acad Sci U S A. 1983;80: 2390–2394. 6572982 10.1073/pnas.80.8.2390 PMC393826

[11] Hidalgo A, Barami K, Iversen K, Goldman SA. Estrogens and non-estrogenic ovarian influences combine to promote the recruitment and decrease the turnover of new neurons in the adult female canary brain. J Neurobiol. 1995;27: 470–487. 10.1002/neu.480270404 7561828

[12] Larson T a Wang T-W, Gale SD Miller KE, Thatra NM Caras ML, et al Postsynaptic neural activity regulates neuronal addition in the adult avian song control system. Proc Natl Acad Sci USA. 2013;110: 16640–4. 10.1073/pnas.1310237110 24062453 PMC3799304

[13] Cattan A, Ayali A, Barnea A. The Cell Birth Marker BrdU Does Not Affect Recruitment of Subsequent Cell Divisions in the Adult Avian Brain. Biomed Res Int. 2015;2015: 1–11. 10.1155/2015/126078 PMC4339786 25759813

[14] Balthazart J, Ball GF. Doublecortin is a highly valuable endogenous marker of adult neurogenesis in canaries. Commentary on Vellema M et al. (2014): Evaluating the predictive value of doublecortin as a marker for adult neurogenesis in canaries (S*erinus canaria*). J Comparative. Brain Behav Evol. 2014;84: 1–4. 10.1159/000362917 25034511 PMC4149834

[15] Barker JM, Charlier TD, Ball GF, Balthazart J. A New Method for In Vitro Detection of Bromodeoxyuridine in Serum: A Proof of Concept in a Songbird Species, the Canary. PLoS One. 2013;8: 1–5. 10.1371/journal.pone.0063692 PMC3653834 23691086

[16] Yamaguchi M, Mori K. Critical period for sensory experience-dependent survival of newly generated granule cells in the adult mouse olfactory bulb. Proc Natl Acad Sci USA. 2005;102: 9697–702. 10.1073/pnas.0406082102 15976032 PMC1157102

[17] Taupin P. BrdU immunohistochemistry for studying adult neurogenesis: paradigms, pitfalls, limitations, and validation. Brain Res Rev. 2007;53: 198–214. 10.1016/j.brainresrev.2006.08.002 17020783

[18] Balthazart J, Boseret G, Konkle ATM, Hurley LL, Ball GF. Doublecortin as a marker of adult neuroplasticity in the canary song control nucleus HVC. Eur J Neurosci. 2008;27: 801–817. 10.1111/j.1460-9568.2008.06059.x 18333960

[19] Balthazart J, Ball GF. Endogenous versus exogenous markers of adult neurogenesis in canaries and other birds: Advantages and disadvantages. J Comp Neurol. 2014/08/19. 2014;522: 4100–4120. 10.1002/cne.23661 25131458 PMC4198497

[20] Boseret G, Ball GF, Balthazart J. The microtubule-associated protein doublecortin is broadly expressed in the telencephalon of adult canaries. J Chem Neuroanat. 2007;33:140–154. 10.1016/j.jchemneu.2007.02.002 17367992 PMC2040224

[21] Francis F, Koulakoff A, Boucher D, Chafey P, Schaar B, Vinet MC, et al Doublecortin is a developmentally regulated, microtubule-associated protein expressed in migrating and differentiating neurons. Neuron. 1999;23: 247–256. 10399932 10.1016/s0896-6273(00)80777-1

[22] Bai J, Ramos RL, Ackman JB, Thomas AM, Lee R V, LoTurco JJ. RNAi reveals doublecortin is required for radial migration in rat neocortex. Nat Neurosci. 2003;6: 1277–1283. 10.1038/nn1153 14625554

[23] Jin K, Mao XO, Cottrell B, Schilling B, Xie L, Row RH, et al Proteomic and immunochemical characterization of a role for stathmin in adult neurogenesis. FASEB J. 2004;18: 287–299. 10.1096/fj.03-0973com 14769823

[24] Moores CA, Perderiset M, Francis F, Chelly J, Houdusse A, Milligan RA. Mechanism of microtubule stabilization by doublecortin. Mol Cell. 2004;14: 833–839. 10.1016/j.molcel.2004.06.009 15200960

[25] Gleeson JG, Lin PT, Flanagan LA, Walsh CA. Doublecortin is a microtubule-associated protein and is expressed widely by migrating neurons. Neuron. 1999;23: 257–271. 10399933 10.1016/s0896-6273(00)80778-3

[26] Nacher J, Crespo C, McEwen BS. Doublecortin expression in the adult rat telencephalon. Eur J Neurosci. 2001;14: 629–644. 11556888 10.1046/j.0953-816x.2001.01683.x

[27] Lankford K, Cypher C, Letourneau P. Nerve growth cone motility. Curr Opin Cell Biol. 1990;2: 80–85. 2139335 10.1016/s0955-0674(05)80035-3

[28] Gordon-Weeks PR. Control of microtubule assembly in growth cones. J Cell Sci Suppl. 1991;15: 45–49. 1824106 10.1242/jcs.1991.supplement_15.7

[29] Rao MS, Shetty AK. Efficacy of doublecortin as a marker to analyse the absolute number and dendritic growth of newly generated neurons in the adult dentate gyrus. Eur J Neurosci. 2004;19: 234–246. 14725617 10.1111/j.0953-816x.2003.03123.x

[30] Perfito N, Guardado D, Williams TD, Bentley GE. Social cues regulate reciprocal switching of hypothalamic dio2/dio3 and the transition into final follicle maturation in European starlings (*Sturnus vulgaris*). Endocrinology. 2015;156: 694–706. 10.1210/en.2014-1450 25490148

[31] Amy M, Salvin P, Naguib M, Leboucher G. Female signalling to male song in the domestic canary, *Serinus canaria*. R Soc Open Sci. 2015;2: 140196 10.1098/rsos.140196 26064577 PMC4448791

[32] Hinde RA, Steel E. The effect of male song on an estrogen-dependent behavior pattern in the female canary (*Serinus canarius*). Horm Behav. 1976;7: 293–304. 992585 10.1016/0018-506x(76)90035-0

[33] Cuthill I, Hindmarsh A. Increase in starling song activity with removal of mate. Anim Behav. 1985;33: 326–328.

[34] Catchpole CK. The Functions of Advertising Song in the Sedge Warbler (*Acrocephalus schoenobaenus)* and the Reed Warbler (*A*. *scirpaceus*). Behaviour. Brill; 1973;46: 300–320.

[35] Alward BA, Mayes WD, Peng K, Stevenson TJ, Balthazart J, Ball GF. Dissociable effects of social context on song and doublecortin immunoreactivity in male canaries. Eur J Neurosci. 2014;40: 2941–2947. 10.1111/ejn.12658 24974859 PMC4528916

[36] Krebs JR, Avery M, Cowie RJ. Effect of removal of mate on the singing behaviour of great tits. Anim Behav. 1981;29: 635–637.

[37] Li XC, Jarvis ED, Alvarez-Borda B, Lim DA, Nottebohm F. A relationship between behavior, neurotrophin expression, and new neuron survival. Proc Natl Acad Sci USA. 2000;97: 8584–8589. 10.1073/pnas.140222497 10890902 PMC26991

[38] Alvarez-Borda B, Nottebohm F. Gonads and singing play separate, additive roles in new neuron recruitment in adult canary brain. J Neurosci. 2002;22: 8684–8690. 12351743 10.1523/JNEUROSCI.22-19-08684.2002 PMC6757767

[39] Boseret G, Carere C, Ball GF, Balthazart J. Social context affects testosterone-induced singing and the volume of song control nuclei in male canaries (*Serinus canaria*). J Neurobiol. 2006;66: 1044–1060. 10.1002/neu.20268 16838373

[40] Lipkind D, Nottebohm F, Rado R, Barnea A. Social change affects the survival of new neurons in the forebrain of adult songbirds. Behav Brain res. 2002;133: 31–43. 12048172 10.1016/s0166-4328(01)00416-8

[41] Barnea A, Mishal A, Nottebohm F. Social and spatial changes induce multiple survival regimes for new neurons in two regions of the adult brain: An anatomical representation of time? Behav Brain Res. 2006;167: 63–74. 10.1016/j.bbr.2005.08.018 16216348

[42] Adar E, Lotem A, Barnea A. The effect of social environment on singing behavior in the zebra finch (*Taeniopygia guttata*) and its implication for neuronal recruitment. Behav Brain Res. 2008;187: 178–184. 10.1016/j.bbr.2007.09.011 17950475

[43] Belnoue L, Grosjean N, Abrous DN, Koehl M. A Critical Time Window for the Recruitment of Bulbar Newborn Neurons by Olfactory Discrimination Learning. J Neurosci. 2011;31: 1010–1016. 10.1523/JNEUROSCI.3941-10.2011 21248125 PMC6632920

[44] Nottebohm F, Arnold AP. Sexual dimorphism in vocal control areas of the songbird brain. Science. 1976;194: 211–213. 959852 10.1126/science.959852

[45] Odom KJ, Hall ML, Riebel K, Omland KE, Langmore NE. Female song is widespread and ancestral in songbirds. Nat Commun. Nature Publishing Group; 2014;5: 3379 10.1038/ncomms4379 24594930

[46] Nottebohm F. Testosterone triggers growth of brain vocal control nuclei in adult female canaries. Brain Res. 1980;189: 429–436. 7370785 10.1016/0006-8993(80)90102-x

[47] Rasika S, Nottebohm F, Alvarez-Buylla A. Testosterone increases the recruitment and/or survival of new high vocal center neurons in adult female canaries. Proc Natl Acad Sci USA. 1994;91: 7854–7858. 8058723 10.1073/pnas.91.17.7854 PMC44502

[48] Madison FN, Rouse ML, Balthazart J, Ball GF. Reversing song behavior phenotype: Testosterone driven induction of singing and measures of song quality in adult male and female canaries (*Serinus canaria*). Gen Comp Endocrinol. Elsevier Inc.; 2014;215: 61–75. 10.1016/j.ygcen.2014.09.008 25260250 PMC4528960

[49] Hall ZJ, MacDougall-Shackleton SA. Influence of testosterone metabolites on song-control system neuroplasticity during photostimulation in adult european starlings (*Sturnus vulgaris*). PLoS One. 2012;7. 10.1371/journal.pone.0040060 PMC3391231 22792214

[50] Guigueno MF, Sherry DF, MacDougall-Shackleton SA. Sex and seasonal differences in neurogenesis and volume of the song-control system are associated with song in brood-parasitic and non-brood-parasitic icterid songbirds. Dev Neurobiol. 2016; 76:1226–1240. 10.1002/dneu.22385 26898912

[51] Stevenson TJ, Bentley GE, Ubuka T, Arckens L, Hampson E, MacDougall-Shackleton SA. Effects of social cues on GnRH-I, GnRH-II, and reproductive physiology in female house sparrows (*Passer domesticus*). Gen Comp Endocrinol. 2008;156: 385–394. 10.1016/j.ygcen.2008.01.015 18295765

[52] Moore M. Effect of female sexual displays on the endocrine physiology and behavior of male white-crowned sparrows. J Zool Lond. 1983;199: 137–148.

[53] Ponti G, Obernier K, Guinto C, Jose L, Bonfanti L, Alvarez-Buylla A. Cell cycle and lineage progression of neural progenitors in the ventricular-subventricular zones of adult mice. Proc Natl Acad Sci USA. 2013;110: E1045–54. 10.1073/pnas.1219563110 23431204 PMC3600494

[54] Llorens-Martín M, Trejo JL. Multiple birthdating analyses in adult neurogenesis: A line-up of the usual suspects. Front Neurosci. 2011;5: 1–8. 21660291 10.3389/fnins.2011.00076 PMC3107564

[55] Vermeulen A, Eens M, Zaid E, Müller W. Baseline innate immunity does not affect the response to an immune challenge in female great tits (*Parus major*). Behav Ecol Sociobiol. 2016;70: 585–592.

[56] Sartor JJ, Balthazart J, Ball GF. Coordinated and dissociated effects of testosterone on singing behavior and song control nuclei in canaries (*Serinus canaria*). Horm Behav. 2005;47: 467–476. 10.1016/j.yhbeh.2004.12.004 15777813

[57] Sartor JJ, Balthazart J, Ball GF. Coordinated and dissociated effects of testosterone on singing behavior and song control nuclei in canaries (*Serinus canaria*). Horm Behav. 2005;47: 467–476. 10.1016/j.yhbeh.2004.12.004 15777813

[58] Appeltants D, Ball GF, Balthazart J. Song activation by testosterone is associated with an increased catecholaminergic innervation of the song control system in female canaries. Neuroscience. 2003;121: 801–814. 14568038 10.1016/s0306-4522(03)00496-2

[59] Alward B a, Balthazart J, Ball GF. Differential effects of global versus local testosterone on singing behavior and its underlying neural substrate. Proc Natl Acad Sci USA. 2013;110: 19573–19578. 10.1073/pnas.1311371110 24218603 PMC3845175

[60] Leboucher G, Kreutzer M, Dittami J. Copulation-solicitation displays in female canaries (*Serinus canaria*): are oestradiol implants necessary? Ethology. 1994;97: 190–197.

[61] Liboska R, Ligasová A, Strunin D, Rosenberg I, Koberna K. Most Anti-BrdU Antibodies React with 2′-Deoxy-5-Ethynyluridine—The Method for the Effective Suppression of This Cross-Reactivity. PLoS One. 2012;7: 1–10. 10.1371/journal.pone.0051679 PMC3525573 23272138

[62] de Bournonville C, Balthazart J, Ball GF, Cornil CA. Non-ovarian aromatization is required to activate female sexual motivation in testosterone-treated ovariectomized quail. Horm Behav. Elsevier B.V.; 2016;83: 45–59. 10.1016/j.yhbeh.2016.05.011 27189762 PMC4916015

[63] de Bournonville C, Dickens MJ, Ball GF, Balthazart J, Cornil CA. Dynamic changes in brain aromatase activity following sexual interactions in males: where, when and why? Psychoneuroendocrinology. 2013;38: 789–799. 10.1016/j.psyneuen.2012.09.001 22999655 PMC3534822

[64] Dickens MJ, Balthazart J, Cornil CA. Brain Aromatase and Circulating Corticosterone are Rapidly Regulated by Combined Acute Stress and Sexual Interaction in a Sex-Specific Manner. J Neuroendocrinol. 2012;24: 1322–1334. 10.1111/j.1365-2826.2012.02340.x 22612582 PMC3510384

[65] Dickens MJ, Cornil CA, Balthazart J. Acute Stress Differentially Affects Aromatase Activity in Specific Brain Nuclei of Adult Male and Female Quail. Endocrinology. 2011;152: 4242–4251. 10.1210/en.2011-1341 21878510 PMC3199009

[66] Dickens MJ, Cornil CA, Balthazart J. Neurochemical Control of Rapid Stress-Induced Changes in Brain Aromatase Activity. J Neuroendocrinol. 2013;25: 329–339. 10.1111/jne.12012 23253172

[67] Dickens MJ, de Bournonville C, Balthazart J, Cornil CA. Relationships between rapid changes in local aromatase activity and estradiol concentrations in male and female quail brain. Horm Behav. 2014;65: 154–164. 10.1016/j.yhbeh.2013.12.011 24368290 PMC3932376

[68] Barami K, Iversen K, Furneaux H, Goldman SA. Hu protein as an early marker of neuronal phenotypic differentiation by subependymal zone cells of the adult songbird forebrain. J Neurobiol. 1995;28: 82–101. 10.1002/neu.480280108 8586967

[69] Vellema M, Ko MC, Frankl-Vilches C, Gahr M. What makes a marker a good marker?. Commentary on Balthazart J and Ball G (2014): Doublecortin is a highly valuable endogenous marker of adult neurogenesis in canaries. Brain Behav Evol 84:1–4. Brain Behav Evol. 2014/07/19. 2014;84: 5–7. 10.1159/000363125 25034769

[70] Balthazart J, Ball GF. Endogenous versus exogenous markers of adult neurogenesis in canaries and other birds: Advantages and disadvantages. J Comp Neurol. 2014;522: 4100–4120. 10.1002/cne.23661 25131458 PMC4198497

[71] Chehrehasa F, Meedeniya ACB, Dwyer P, Abrahamsen G, Mackay-Sim A. EdU, a new thymidine analogue for labelling proliferating cells in the nervous system. J Neurosci Methods. 2009;177: 122–130. 10.1016/j.jneumeth.2008.10.006 18996411

[72] Salic A, Mitchison TJ. A chemical method for fast and sensitive detection of DNA synthesis in vivo. Proc Natl Acad Sci USA. 2008/02/15. 2008;105: 2415–2420. 10.1073/pnas.0712168105 18272492 PMC2268151

[73] Warren M, Puskarczyk K, Chapman SC. Chick embryo proliferation studies using EdU labeling. Dev Dyn. 2009;238: 944–949. 10.1002/dvdy.21895 19253396 PMC2664394

[74] Zeng C, Pan F, Jones LA, Lim MM, Griffin EA, Sheline YI, et al Evaluation of 5-ethynyl-2’-deoxyuridine staining as a sensitive and reliable method for studying cell proliferation in the adult nervous system. Brain Res. 2010;1319: 21–32. 10.1016/j.brainres.2009.12.092 20064490 PMC2826567

[75] Yamamura T, Barker JM, Balthazart J, Ball GF. Androgens and Estrogens Synergistically Regulate the Expression of Doublecortin and Enhance Neuronal Recruitment in the Song System of Adult Female Canaries. J Neurosci. 2011;31: 9649–9657. 10.1523/JNEUROSCI.0088-11.2011 21715630 PMC3214644

[76] Chen Q, Zhang X, Zhao Y, Zhou X, Sun L, Zeng S, et al Sexual differences in cell proliferation in the ventricular zone, cell migration and differentiation in the HVC of juvenile bengalese finch. PLoS One. 2014;9: 1–18. 10.1371/journal.pone.0097403 PMC4026142 24841082

[77] Scott BB, Lois C. Developmental origin and identity of song system neurons born during vocal learning in songbirds. J Comp Neurol. 2007;502: 202–214. 10.1002/cne.21296 17348018

[78] Vellema M, van der Linden A, Gahr M. Area-specific migration and recruitment of new neurons in the adult songbird brain. J Comp Neurol. 2010/02/27. 2010;518: 1442–1459. 10.1002/cne.22281 20187140

[79] Leitner S, Catchpole CK. Song and brain development in canaries raised under different conditions of acoustic and social isolations over two years. 2007;67:1478–1487. 10.1002/dneu.20521 17525993

[80] Balthazart J, Boseret G, Konkle AT, Hurley LL, Ball GF. Doublecortin as a marker of adult neuroplasticity in the canary song control nucleus HVC. Eur J Neurosci. 2008;27: 801–817. 10.1111/j.1460-9568.2008.06059.x 18333960

[81] Delville Y, Sulon J, Hendrick JC, Balthazart J. Effect of the presence of females on the pituitary-testicular activity in male Japanese quail (*Coturnix coturnix japonica*). Gen Comp Endocrinol. 1984;55: 295–305. 6479577 10.1016/0016-6480(84)90115-1

[82] Morton ML, Pereyra ME, Baptista LF. Photoperiodically induced ovarian growth in the white-crowned sparrow (*Zonotrichia leucophrys gambelii*) and its augmentation by song. Comp Biochem Physiol A. 1985;80A: 93–97.

[83] Ball GF, Balthazart J. Neuroendocrine mechanisms regulating reproductive cycles and reproductive behavior in birds. Horm Brain Behav. 2002;2: 649–798.

[84] Brown SD, Johnson F, Bottjer SW. Neurogenesis in adult canary telencephalon is independent of gonadal hormone levels. J Neurosci. 1993;13: 2024–2032. 8478689 10.1523/JNEUROSCI.13-05-02024.1993 PMC6576574

[85] Rasika S, Nottebohm F, Alvarez-Buylla a. Testosterone increases the recruitment and/or survival of new high vocal center neurons in adult female canaries. Proc Natl Acad Sci USA. 1994;91: 7854–7858. 8058723 10.1073/pnas.91.17.7854 PMC44502

[86] Barker JM, Ball GF, Balthazart J. Anatomically discrete sex differences and enhancement by testosterone of cell proliferation in the telencephalic ventricle zone of the adult canary brain. J Chem Neuroanat. 2014;55: 1–8. 10.1016/j.jchemneu.2013.10.005 24211440 PMC3951581

[87] Barker JM, Ball GF, Balthazart J. Sex differences in proliferation and maturation-rates of adult generated neurons in canaries. Abstracts of the FENS meeting, Barcelona SP 2012.

[88] Gould E, Cameron HA a, Daniels DCC, Woolley CSS, McEwen BSS. Adrenal hormones suppress cell division in the adult rat dentate gyrus. J Neurosci. 1992;12: 3642–3650. 1527603 10.1523/JNEUROSCI.12-09-03642.1992 PMC6575731

[89] Newman AEM, MacDougall-Shackleton SA, An Y-S, Kriengwatana B, Soma KK. Corticosterone and dehydroepiandrosterone have opposing effects on adult neuroplasticity in the avian song control system. J Comp Neurol. United States; 2010;518: 3662–3678. 10.1002/cne.22395 20653028

[90] Katz A, Mirzatoni A, Zhen Y, Schlinger BA. Sex differences in cell proliferation and glucocorticoid responsiveness in the zebra finch brain. Eur J Neurosci. 2008;28: 99–106. 10.1111/j.1460-9568.2008.06303.x 18662338 PMC2678882

[91] Alvarez-Buylla A, Kirn JR. Birth, migration, incorporation, and death of vocal control neurons in adult songbirds. J Neurobiol. 1997;33: 585–601. 9369461

[92] Nottebohm F. The road we travelled: discovery, choreography, and significance of brain replaceable neurons. Ann N Y Acad Sci. 2004;1016: 628–658. 10.1196/annals.1298.027 15313798

[93] Nottebohm F. The neural basis of birdsong. PLoS Biol. 2005;3: e164 10.1371/journal.pbio.0030164 15884976 PMC1110917

[94] Brenowitz EA. Plasticity of the song control system in adult birds In: Zeigler HP, Marler P, editors. Neuroscience of birdsong. Cambridge: Cambridge University Press; 2008 pp. 332–349.

[95] Chen Z, Ye R, Goldman SA. Testosterone modulation of angiogenesis and neurogenesis in the adult songbird brain. Neuroscience. 2013;239: 139–148. 10.1016/j.neuroscience.2012.12.043 23291451 PMC4113966

